# Aberrant expression of agouti signaling protein (*ASIP*) as a cause of monogenic severe childhood obesity

**DOI:** 10.1038/s42255-022-00703-9

**Published:** 2022-12-19

**Authors:** Elena Kempf, Kathrin Landgraf, Robert Stein, Martha Hanschkow, Anja Hilbert, Rami Abou Jamra, Paula Boczki, Gunda Herberth, Andreas Kühnapfel, Yu-Hua Tseng, Claudia Stäubert, Torsten Schöneberg, Peter Kühnen, N. William Rayner, Eleftheria Zeggini, Wieland Kiess, Matthias Blüher, Antje Körner

**Affiliations:** 1 University Hospital for Children and Adolescents, Center for Pediatric Research, Medical Faculty, University of Leipzig, Leipzig, Germany; 2 Helmholtz Institute for Metabolic Obesity and Vascular Research (HI-MAG) of the Helmholtz Zentrum München at the University of Leipzig and University Hospital Leipzig, Leipzig, Germany; 3 Department of Psychosomatic Medicine and Psychotherapy, Medical Faculty, University of Leipzig, Leipzig, Germany; 4 University Medical Center Leipzig, Institute of Human Genetics, Medical Faculty, University of Leipzig, Leipzig, Germany; 5 Department of Environmental Immunology, Helmholtz Centre for Environmental Research-UFZ, Leipzig, Germany; 6 Institute for Medical Informatics, Statistics and Epidemiology, Medical Faculty, University of Leipzig, Leipzig, Germany; 7 Section on Integrative Physiology and Metabolism, Joslin Diabetes Center, Harvard Medical School, Boston, MA USA; 8 Division of Molecular Biochemistry, Rudolf Schönheimer Institute of Biochemistry, Medical Faculty, University of Leipzig, Leipzig, Germany; 9 Institute for Experimental Pediatric Endocrinology, Charité Universitätsmedizin Berlin, Corporate Member of Freie Universität Berlin, Humboldt-Universität zu Berlin, Berlin, Germany; 10 Institute of Translational Genomics, Helmholtz Zentrum München - German Research Center for Environmental Health, Neuherberg, Germany; 11 TUM School of Medicine, Translational Genomics, Technical University of Munich and Klinikum Rechts der Isar, Munich, Germany; 12 LIFE–Leipzig Research Center for Civilization Diseases, Medical Faculty, University of Leipzig, Leipzig, Germany; 13 Medical Department III-Endocrinology, Nephrology, Rheumatology, University of Leipzig, Leipzig, Germany

**Keywords:** Obesity, Paediatric research, Metabolic syndrome, Gene regulation, Metabolism

## Abstract

Here we report a heterozygous tandem duplication at the *ASIP* (agouti signaling protein) gene locus causing ubiquitous, ectopic *ASIP* expression in a female patient with extreme childhood obesity. The mutation places *ASIP* under control of the ubiquitously active itchy E3 ubiquitin protein ligase promoter, driving the generation of ASIP in patient-derived native and induced pluripotent stem cells for all germ layers and hypothalamic-like neurons. The patient’s phenotype of early-onset obesity, overgrowth, red hair and hyperinsulinemia is concordant with that of mutant mice ubiquitously expressing the homolog nonagouti. ASIP represses melanocyte-stimulating hormone-mediated activation as a melanocortin receptor antagonist, which might affect eating behavior, energy expenditure, adipocyte differentiation and pigmentation, as observed in the index patient. As the type of mutation escapes standard genetic screening algorithms, we rescreened the Leipzig Childhood Obesity cohort of 1,745 patients and identified four additional patients with the identical mutation, ectopic *ASIP* expression and a similar phenotype. Taken together, our data indicate that ubiquitous ectopic *ASIP* expression is likely a monogenic cause of human obesity.

## Abstract (web summary)

A mutation leading to ubiquitous ectopic expression of agouti signaling protein, which is not picked up in conventional genetic screenings, is reported in patients with severe childhood obesity.

## Main

Although there is doubtless a genetic component of common obesity, monogenic forms of human obesity are very rare^1,2^. Most of these single-gene defects affect the leptin–melanocortin 4 receptor (MC4R) pathway, which centrally regulates food intake and energy balance^3,4^. Detection of these^5^ and new monogenic obesity traits^6^ will not only improve our understanding of obesity mechanisms in humans but guide the development of new targeted pharmacotherapies increasingly available for affected patients^2,7^.

In a bottom-up approach, taking advantage of patient-derived adipose cells, we identified a potential new genetic cause of melanocortin obesity syndrome, phenocopying mouse and human proopiomelanocortin (*POMC*) deficiency and mouse *ASIP* mutants (so-called ‘agouti mice’).

## Case report

**Fig. 1:**
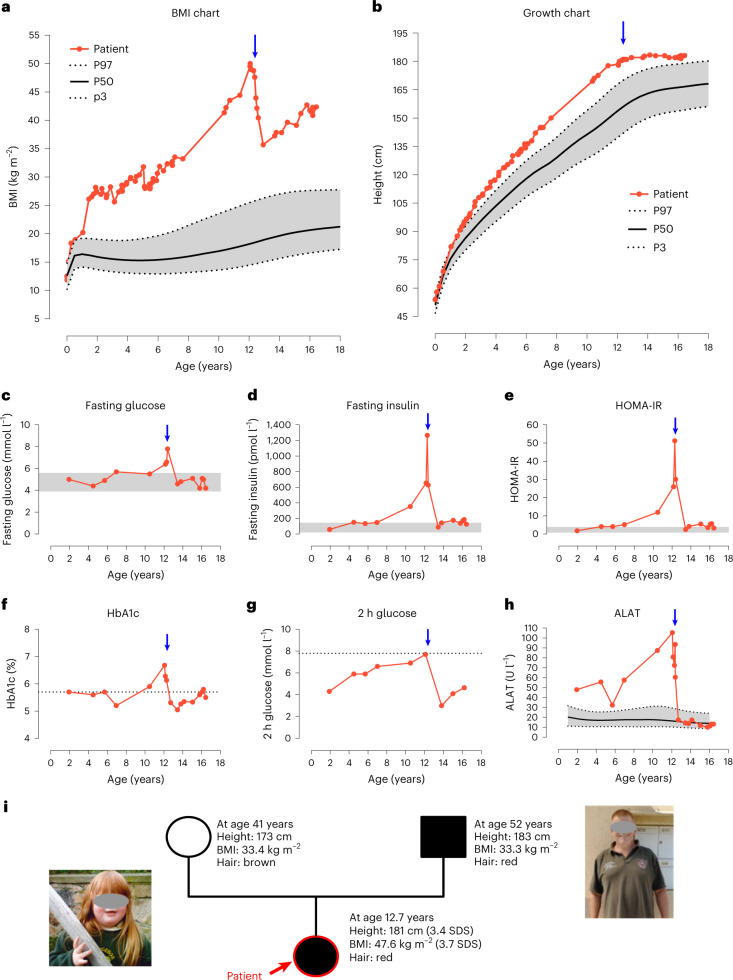
Clinical characteristics of the index patient and her family. **a**,**b**, Course of BMI (**a**) and height (**b**) of the patient (red dots) from birth to adolescence in relation to 3rd (P3), 50th (P50) and 97th (P97) sex-specific reference percentiles according to Kromeyer-Hauschild et al.^40^. Data from the patient were corrected for gestational age until the age of 2 years. **c**–**g**, Parameters of glucose metabolism with the respective reference ranges of either the local hospital laboratory depicted as gray areas or with prediabetes cut-offs according to the American Diabetes Association^65^ indicated by a dotted line. **h**, Serum levels of liver enzyme alanine aminotransferase (ALAT) in comparison to the reference values according to Bussler et al. ^66^ (shaded in gray). Blue arrows mark the time point of bariatric surgery at the age of 12.4 years. **i**, Pedigree of the family; females are indicated by circles, the father by a square. Individuals carrying the *ASIP* tandem duplication are indicated by black symbols, the open symbol indicates the mother not carrying the mutation. The index patient is indicated by a red arrow. Photographs present patient at age of 3.5 years and father at age 52 years. HbA1c, glycated hemoglobin; HOMA-IR, homeostatic model assessment for insulin resistance; 2 h glucose, glucose levels after 2 h of oral glucose tolerance testing. Source data

A girl of non-consanguineous parents of European ancestry first presented at the age of 1.9 years with overgrowth and severe obesity. From infancy onwards, she was reported to have a constant desire for food and developed progressive extreme obesity and tall stature (Fig. 1a,b) accompanied by first signs of severe hyperinsulinemia and hepatic steatosis in early childhood (Fig. 1c–h).

Both parents also suffer from obesity (Fig. 1i). The father has type 2 diabetes, hypertension and gout. In distinct phenotypic features (red hair color, pale skin and freckles), the girl resembles her father. Clinically relevant variants in genes related to monogenic obesity and other inherited diseases were excluded by whole exome sequencing. At the age of 12.4 years with a body mass index (BMI) of 47.6 kg m^−^^2^ (BMI s.d. score (SDS) 3.8, weight 156 kg, height 181 cm and 3.34 SDS), she underwent bariatric surgery by sleeve gastrectomy leading to weight loss of 40 kg. She subsequently regained weight although metabolic improvement was retained (Fig. 1a–h).

During childhood, the patient reported being continuously hungry, except after eating very large amounts of food. She tried consciously to control food intake. At the age of 16 years, she scored high on the Eating Disorder Examination Questionnaire and the Dutch Eating Behavior Questionnaire, showing high cognitive restraint (>85th percentile) as well as extreme eating, weight and shape concerns (>95th percentile). She did not report binge eating, external (<5th percentile) or emotional eating (<25th percentile).

A clinical–psychological exploration noted remarkably low physical activity. Furthermore, indirect calorimetry revealed a reduced resting metabolic rate (1,660 kcal d^−1^, accounting for only 77.4% of the expected).

Hence, this phenotype of early-onset extreme obesity accompanied by tall stature, hypopigmentation and alterations in appetite and energy expenditure was suggestive for an underlying genetic or biological cause.

## Results

### Biological alterations and ectopic *ASIP* expression in adipose tissue

**Fig. 2:**
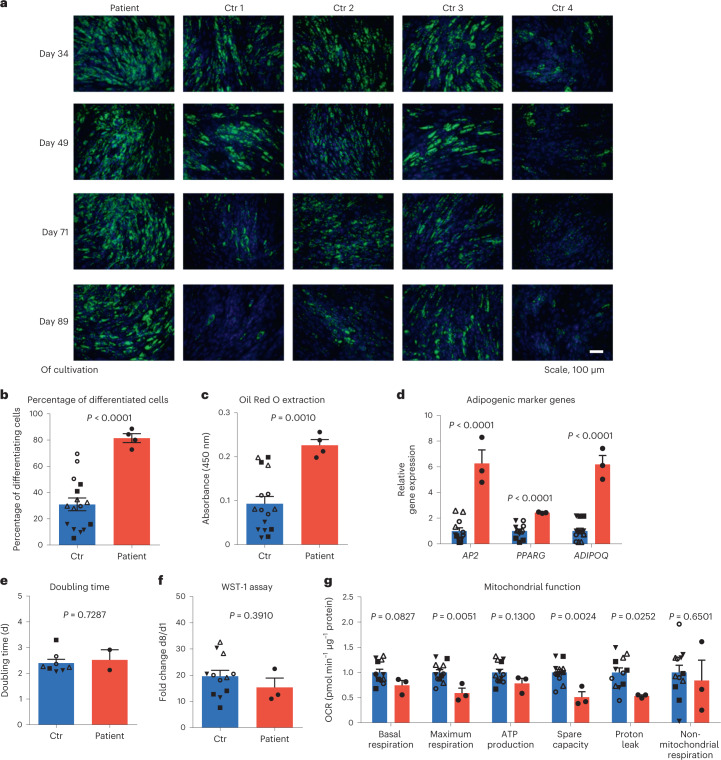
Adipogenic and mitochondrial function of patient-derived adipose cells. **a**, At indicated time points of cultivation, subcutaneous SVF cells from the patient and control (Ctr) children, all presenting with overweight or obesity (*n* = 4; 3 females, 1 male), matched for age (mean age 13.8 years) were differentiated into adipocytes. Nuclei were stained with Hoechst (blue) and lipids with Nile red (green). **b**, Percentage of differentiated cells determined by cell counting. **c**, Using the same differentiated cells adipogenic capacity was quantified using Oil Red O. **d**, Relative expression of the adipogenic marker genes adipocyte protein 2 (*AP2*), peroxisome proliferator-activated receptor γ (*PPARG*) and adiponectin (*ADIPOQ*) in differentiated SVF cells from the patient and controls (*n* = 4). The differentiation was examined over *n* = 3 independent experiments (cells seeded for differentiation at days 65, 83 and 98 of cultivation). Data are presented as fold change in relation to the controls. **e**, Doubling time of SVF cells from the patient and control children (Ctr, *n* = 4) shown as mean with s.e. from *n* = 2 experiments at day 49 and 71 of cultivation. **f**, Cell viability was assessed by WST-1 assay in *n* = 3 experiments at day 49, 71 and 89 of cultivation. **g**, Mitochondrial function of SVF cells from the patient and controls (*n* = 4) measured in *n* = 3 independent experiments using the Seahorse Mito Stress Test kit. Data are presented as fold change in relation to the controls. The data points of each independent experiment are indicated for each individual (**b**–**g**). Bar plots represent the mean ± s.e.m. All bar plots from controls are shown in blue and from the patient in red. Statistical significance was assessed using two-sided Student’s *t*-test without adjustment of multiple comparisons. Source data

We obtained a sample of subcutaneous adipose tissue from the patient during bariatric surgery. Considering the severe overgrowth and obesity, we hypothesized that the adipose tissue-derived stromal vascular fraction (SVF) cells showed alterations in proliferative and/or adipogenic capacity. The patient’s SVF cells showed pronounced adipocyte differentiation that was sustained in long-term cultivation compared to SVF cells from four age-matched controls with obesity (Supplementary Appendix, Supplementary Table 1 and Fig. 2a–d), whereas proliferation of the patient’s SVF cells was not enhanced (Fig. 2e,f). We further found reduced mitochondrial maximum respiration, spare capacity and proton leak in the patient’s SVF cells as proxies for reduced energy expenditure (Fig. 2g).

**Fig. 3:**
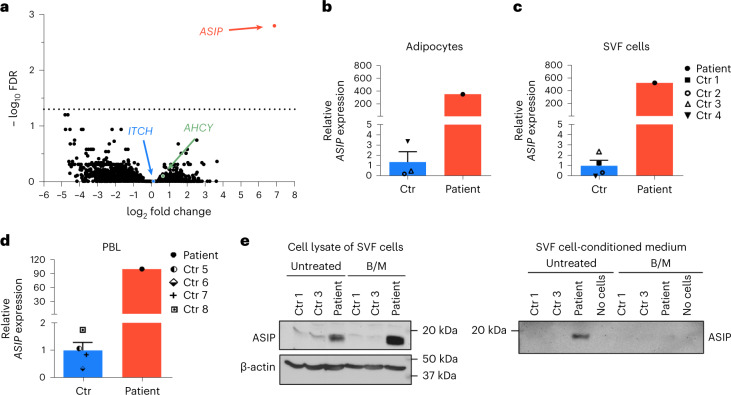
Identification of ectopic *ASIP* expression in the patient. **a**, Differentially expressed genes between undifferentiated SVF cells from the patient and control children (Ctr; *n* = 4) presented as the log_2_ fold change of gene expression across −log_10_ false discovery rate (FDR). Similar results have been shown in a second independent experiment. **b**–**d**, *ASIP* gene expression levels are shown for adipocytes (**b**), SVF cells (**c**) and peripheral blood leukocytes (PBLs) (**d**). As PBL RNA samples from control children 1–4 were not available, measurements were performed in control children 5–8 (Supplementary Appendix and Supplementary Table 1). **e**, ASIP protein was detected by immunoblotting in cell lysates and conditioned medium from untreated and brefeldin A and monensin (B/M)-treated SVF cells from the patient and two control children, 1 and 3. Similar results were obtained by a second independent experiment. Gene expression data shown in **b**–**d** were normalized to the expression of β-actin and TATA-box-binding protein. Bar plots represent mean ± s.e.m. For **b**–**d** the fold changes in relation to mean of controls ± s.e.m. are indicated. All bar plots from controls are shown in blue and from the patient in red. Source data

To identify potential molecular causes for the enhanced adipogenesis, we searched for differentially expressed genes in the patient’s SVF cells compared to SVF cells from control children through transcriptome-wide analyses. Only one gene encoding *ASIP* was highly overexpressed in the index patient’s cells (Fig. 3a).

We confirmed ectopic *ASIP* expression in several cell types, including isolated adipocytes, SVF cells and peripheral blood leukocytes, indicating ubiquitous expression in the patient (Fig. 3b–d, Supplementary Appendix and Supplementary Table 2). Concordantly, we found ectopic ASIP protein in SVF cell lysates and conditioned medium in the patient’s cells. Inhibition of the secretory pathway lead to cellular retention of ASIP, confirming that the ectopically expressed ASIP is secreted^8^ (Fig. 3e).

Physiologically, *ASIP* is expressed in the skin and regulates hair pigmentation through interaction with the melanocortin-1 receptor (MC1R). Ubiquitous expression of the murine *ASIP* homolog nonagouti in so-called agouti mice leads to yellow fur and obesity due to increased food intake, lipid storage in adipose tissue and reduced energy expenditure^9–12^. Hence, the concordant phenotype associated with ectopic expression of *ASIP* in our participants is likely a new monogenic obesity cause in humans.

### Chromosomal rearrangement causes aberrant ectopic *ASIP* expression

**Fig. 4:**
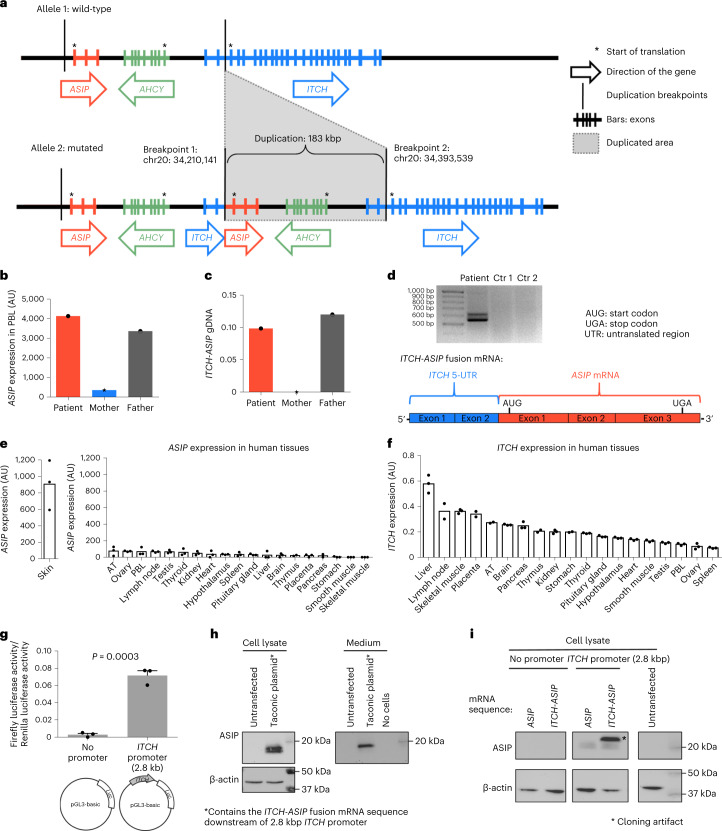
Chromosomal rearrangement at the *ASIP* locus driving ectopic *ASIP* expression. **a**, Schematic of the wild-type allele and the 183-kbp tandem duplication at the *ASIP*, *AHCY* and *ITCH* locus found in the patient. Locations of the breakpoints are presented according to the Dec. 2013 GRCh38/hg38 assembly. **b**, *ASIP* expression in PBLs in the patient, mother and father normalized to β-actin and *TBP*. **c**, Quantification of breakpoint-specific *ITCH*–*ASIP* fusion genomic DNA (gDNA) copy number in the patient, mother and father normalized to the copy number of β-actin gDNA. **d**, 5′-RACE–PCR from the patient and *n* = 2 controls (Ctr). The lower band contained the *ITCH*–*ASIP* fusion mRNA sequence depicted below. The upper bands are incompletely spliced mRNA variants. **e**,**f**, *ASIP* (**e**) and *ITCH* (**f**) expression across human tissues normalized to expression of β-actin and *TBP*. Tissue RNA pooled from several individuals was purchased TakaraBio (three technical replicates). **g**, Luciferase assays in HEK293 cells transfected with vectors with or without a 2.8-kbp *ITCH* promoter sequence upstream of the luciferase (Luc) reporter gene. Firefly Luc activity was normalized to *Renilla* Luc activity. Bar plots represent mean ± s.e.m. of *n* = 3 independent experiments. Statistical significance was assessed using two-sided Student’s *t*-test. **h**, Immunoblot of ASIP in cell lysates and supernatants from HEK293 cells transfected with the Taconic plasmid containing the *ITCH*–*ASIP* mRNA sequence under control of the 2.8-kbp *ITCH* promoter. **i**, Immunoblot of ASIP in cell lysates and supernatants from HEK293 cells transfected with plasmids containing the physiological *ASIP* coding sequence or the *ITCH*–*ASIP* fusion mRNA sequence downstream of no promoter or the *ITCH* promoter. Asterisk indicates a cloning artifact as an additional band due to introduction of an additional translation start codon upstream of the mRNA sequence from the restriction enzyme *Nco*I (C’CATGG). This generated a functional open reading frame, exclusively for the *ITCH*–*ASIP* mRNA sequence, leading to an ASIP protein with additional N-terminal amino acids. The results shown in **h**,**i** were each confirmed by a second independent experiment. Source data

Based on our finding of ectopic *ASIP* expression in the patient, we hypothesized an *ASIP* gene locus alteration. Genetic analyses including trio whole-genome sequencing (WGS), screen for copy-number variation (CNV) and cloning of the breakpoint sequence revealed a heterozygous 183-kbp tandem duplication on chromosome 20 encompassing the neighboring genes *ASIP* and itchy E3 ubiquitin protein ligase *(ITCH*) on the forward strand and adenosylhomocysteinase *(AHCY)* on the reverse strand (Fig. 4a). Additional clinically relevant variants or CNVs in genes related to obesity, such as *MC4R*, *POMC* and *LEPR*, were excluded by exome sequencing and a TruSight One Sequencing panel.

**Extended Data Fig. 1:**
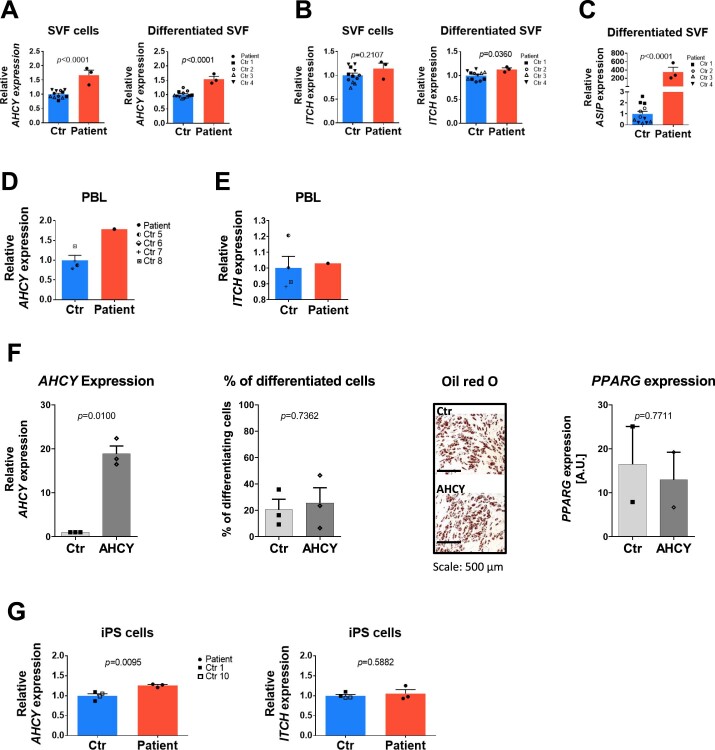
*AHCY* overexpression in different cell types. Gene expression levels of (A) adenosylhomocysteinase (*AHCY*) and (B) itchy E3 ubiquitin protein ligase (*ITCH*) in stromal vascular fraction (SVF) cells before and after adipocyte differentiation and compared to that of (C) agouti signaling protein (*ASIP*) in differentiated SVF cells are shown from the patient and controls (Ctr) patients 1-4 (n=4; 3 females, 1 male) matched for age (mean age 13.8 years) and body weight state (all presenting with overweight or obesity). Data points of n=3 independent experiments are indicated for each subject. In peripheral blood leukocytes (PBL) *AHCY* (D) and *ITCH* (E) expression are shown from the patient and controls. Data points are indicated for each subject. (F) Relative *AHCY* expression in Simpson–Golabi-Behmel syndrome (SGBS) cells after transfection with pCMV-Entry control vector (Ctr) or pCMV-AHCY (AHCY) in n=3 independent experiments. Transfected SGBS cells were differentiated into adipocytes and the percentage of differentiated cells was determined by cell counting after Hoechst and Nile red staining. Using the same cells adipogenic capacity was compared by Oil red O staining (representative microscopic pictures from one of three independent experiments; scale bar = 500µM) and gene expression of the adipogenic marker gene peroxisome proliferator-activated receptor gamma (*PPARG*) after differentiation in n=2 independent experiments was determined. Bar plots show the mean ± SEM. (G) *AHCY* and *ITCH* expression levels are shown for SVF cell-derived induced pluripotent stem cells (iPSCs) from three clones of the patient and two clones from each of the control (Ctr) children, 1 and 10. Data points for each clone from each subject are indicated. The bar plots A-E and G present the mean values ± SEM as a fold change of the controls. Gene expression data were normalized to the expression of β-actin and TATA-box-binding protein. All bars from controls are shown in blue and from the patient in red. *P* values derived using two-sided Student’s t-test are given. Source data

Both, *AHCY* and *ITCH* were not differentially regulated in transcriptome-wide analyses (Fig. 3a) and results from quantitative real-time PCR from SVF cells showed an approximately 1.5-fold increase in *AHCY* expression and no difference in *ITCH* expression (Extended Data Fig. 1a,b), whereas *ASIP* is overexpressed several hundredfold (Extended Data Fig. 1c). Similar results were obtained for peripheral blood leukocytes (Fig. 3d and Extended Data Fig. 1d,e). This is in line with the expectation from the genomic rearrangement resulting in three copies of the *AHCY* gene, no change in the number of *ITCH* coding exons and a switch of promoter usage driving *ASIP* gene expression by the ubiquitously active *ITCH* promoter.

As we saw a slight increase in *AHCY* expression, we investigated the effects of AHCY in more detail; however, experimental overexpression of *AHCY* in human Simpson–Golabi–Behmel syndrome (SGBS) preadipocytes^13^ did not affect adipocyte differentiation or the expression of peroxisome proliferator-activated receptor-γ (*PPARG*) as the master regulator of adipogenesis (Extended Data Fig. 1f). Hence, the main consequence of the chromosomal rearrangement mutation is ectopic *ASIP* expression.

Our index patient inherited the mutation from her father, as evidenced by ectopic *ASIP* expression in his peripheral blood leukocytes (Fig. 4b) and the presence of the duplication-specific *ITCH*–*ASIP* breakpoint sequence confirmed by WGS and breakpoint-specific quantitative polymerase chain reaction (qPCR) (Fig. 4c). The father harbors the same chromosomal rearrangement and according to self-reports had been taller and heavier than his peers from birth onwards. Starting at the age of 10 years, he engaged in competitive athletic sports and lost some weight. Nonetheless, he reported weighing 125 kg at the age of 18 years (BMI 36 kg m^−2^). He did not, however, score conspicuously on eating behavior questionnaires at the age of 52 years.

Both the patient and her father have red hair color and pale skin with freckles (Fig. 1i), which is another characteristic concordant with the yellow fur of the agouti mice ectopically expressing *ASIP* and is due to the action of ASIP as an antagonist at MC1R in the skin. We verified that neither the patient nor the father carries any genetic variant known to be associated with red hair, for example in *MC1R*^14^ (Supplementary Appendix and Supplementary Table 3).

By 5′ rapid amplification of complementary DNA ends and PCR (5′-RACE–PCR) and RNA-seq from peripheral blood and SVF cells, we demonstrated that the genetic rearrangement creates an *ITCH*–*ASIP* transcript consisting of the first two non-coding *ITCH* exons (5′ untranslated region (UTR)) fused to the *ASIP* coding exons (Fig. 4d). This indicates that *ASIP* expression is under the control of the *ITCH* promoter. Concordant with the GTEx catalog, we found high *ASIP* gene expression in skin and low expression in other healthy human tissues (Fig. 4e). As *ITCH* is ubiquitously expressed in human tissues (Fig. 4f and concordant with the GTEx catalog), it explains the ubiquitous expression of *ASIP* in our patient.

We verified functional *ITCH*–*ASIP* interaction in luciferase reporter assays showing that a 2.8-kbp genomic region located upstream of *ITCH* exon 1 is capable of driving gene expression (Fig. 4g). Furthermore, we generated an artificial fusion gene by cloning the *ITCH*–*ASIP* fusion cDNA sequence under control of the 2.8-kbp *ITCH* promoter and confirmed increased production and secretion of ASIP protein (Fig. 4h) independent of the presence or absence of the *ITCH* 5′-UTR (Fig. 4i).

Thus, the chromosomal rearrangement positioning *ASIP* under the control of the *ITCH* promoter is the cause of the ubiquitous expression of *ASIP* in our patient and may thus be responsible for the clinical phenotype of our patient with early-onset extreme obesity, altered eating behavior, reduced energy expenditure and hypopigmentation.

### Retained ectopic *ASIP* expression in patient-derived iPSCs

**Fig. 5:**
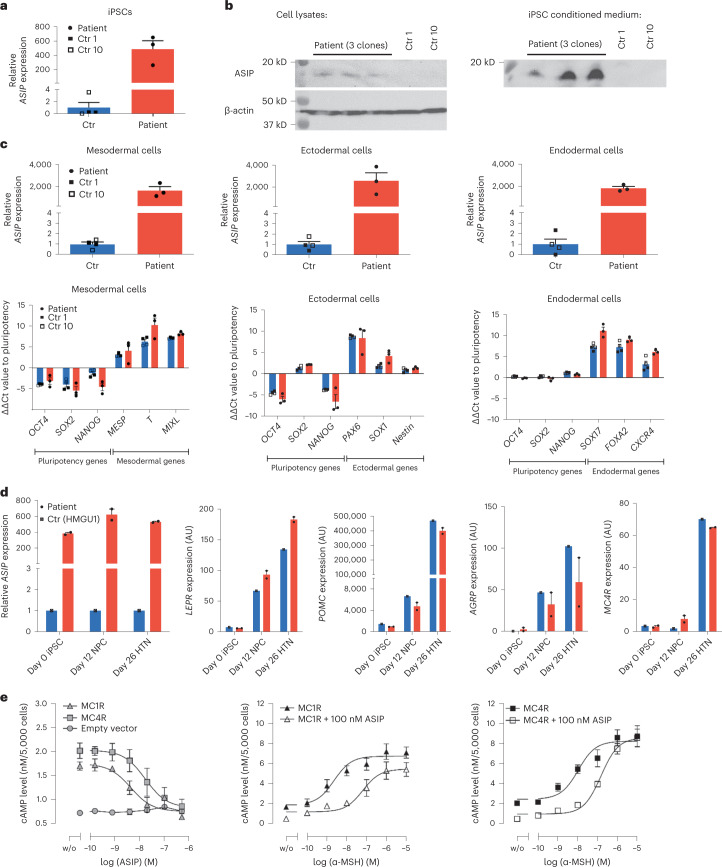
Ectopic *ASIP* expression in patient-derived iPSCs persists during differentiation. **a**, *ASIP* expression in SVF cell-derived iPSCs from *n* = 3 clones (as biological independent samples) of the patient and *n* = 2 clones from each control (Ctr) 1 and 10. **b**, Immunoblot of ASIP in cell lysates and supernatants from untreated iPSCs from the patient and the two control children. Similar results were obtained by a second independent experiment. **c**, Persisting *ASIP* expression in iPSCs of the patient (*n* = 3 clones as biologically independent samples) but not of control children (*n* = 2 clones each) differentiated into the three germ layers. Successful differentiation was confirmed by downregulation of the pluripotency markers *OCT4*, *SOX2* and *NANOG* and upregulation of germ-layer-specific markers (*MESP*, *T* and *MIXL* for mesoderm; *PAX6*, *SOX1* and *Nestin* for ectoderm; *SOX17*, *FOXA2* and *CXCR4* for endoderm). **d**, Persisting *ASIP* expression in patient-derived iPSCs (*n* = 2 clones; red) but not HMGU1 iPSC control cell line (blue) differentiated into hypothalamic-like neurons (HTNs) via neuronal progenitor cells (NPCs). Expression of hypothalamic marker genes *LEPR*, *POMC*, *AGRP* and *MC4R* confirmed successful differentiation. Gene expression levels were normalized to β-actin and TATA-box-binding protein. *ASIP* expression is presented as fold change in relation to the mean ± s.e.m. of the controls. Relative expression levels of germ-layer marker genes were calculated by delta-delta Ct method normalized to β-actin. **e**, Antagonistic activity of ASIP in absence and presence of α-MSH at human MC1R and MC4R. Basal cAMP levels concentration-dependently decreased in presence of increasing concentrations of ASIP (MC1R IC_50_ = 4.4 nM; MC4R IC_50_ = 16.9 nM). α-MSH stimulates both MC1R (EC_50_ = 2.3 nM) and MC4R (EC_50_ = 10.6 nM). Presence of 100 nM ASIP lowers the potency of α-MSH at MC1R (EC_50_ = 65 nM) and MC4R (EC_50_ = 149 nM). Data are mean ± s.e.m. of *n* = 3 independent experiments each performed in duplicate. Source data

To cause this agouti-like phenotype would require *ASIP* expression in central (hypothalamic) circuits. As these are naturally not directly accessible from patient samples and to provide further support that the genetic rearrangement in the patient causes ubiquitous *ASIP* expression in cells of all origins, we generated induced pluripotent stem cells (iPSCs) from patient-derived SVF cells and two control children. Ectopic *ASIP* expression persists in iPSCs from the patient compared to the controls (about 500-fold compared to controls; Fig. 5a), whereas *AHCY* is only slightly increased (about 1.25-fold) and *ITCH* is not differently expressed compared to controls (Extended Data Fig. 1g). We confirmed that ASIP protein is also secreted into the supernatant from patient iPSCs (Fig. 5b). We furthermore differentiated patient and control iPSCs into cells of the three germ layers mesoderm, ectoderm and endoderm and found ectopic *ASIP* expression in patient-derived iPSCs but not controls after differentiation in all germ layers (Fig. 5c). As the suspected mode of action is ectopic ASIP action in the hypothalamus, we went on and confirmed *ASIP* overexpression in patient-derived iPSCs differentiated into hypothalamic-like neurons via neuronal progenitor cells (Fig. 5d).

Finally, to experimentally confirm that ASIP indeed acts as an antagonist not only at MC1R but also at MC4R, we performed cAMP accumulation assays in CHO-K1 cells transiently transfected with human *MC1R* or *MC4R*. Increasing concentrations of ASIP cause a reduction of basal activity of both human MC1R and MC4R activity with half-maximum inhibitory concentration (IC_50_) values of 4.4 nM and 16.9 nM, respectively. The natural ligand α-melanocyte-stimulating hormone (α-MSH) exerted agonistic activity at both receptors (MC1R half-maximum effective concentration (EC_50_) = 2.3 nM; MC4R EC_50_ = 10.6 nM), but presence of 100 nM ASIP caused a reduction of α-MSH potency at both receptors, MC1R (EC_50_ = 65 nM) and MC4R (EC_50_ = 149 nM) (Fig. 5e).

Hence, ubiquitous expression of the nonagouti homolog *ASIP* is found in patient-derived cells of all three germ layers supporting the hypothesis that ectopic ASIP antagonizes MC4R signaling in the hypothalamus of our patient, which would be required to affect processes related to eating behavior and energy expenditure and hence the obesity phenotype.

### Identification of additional patients with *ASIP* mutation

The type of genetic rearrangement is not readily detectable by standard genetic screening classically focusing on single-nucleotide variations. This may explain why there are no cases with detected *ASIP* mutations in the UK Biobank deposit or in the literature yet.

**Fig. 6:**
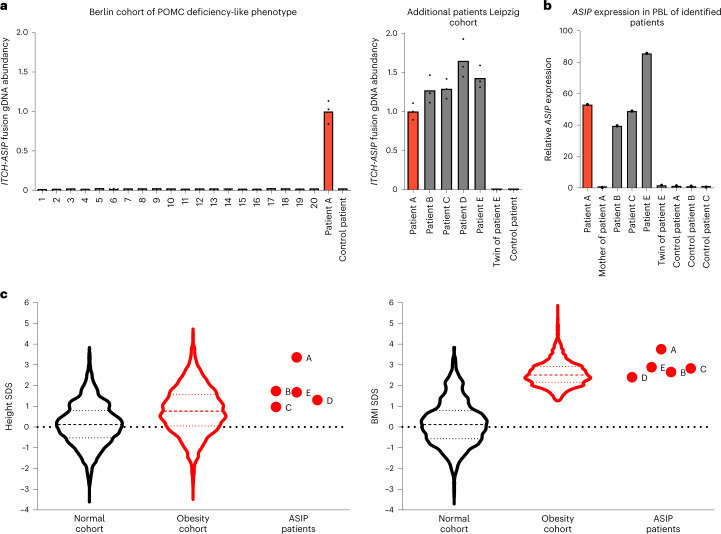
Screening of obesity and populational cohorts for *ASIP* mutations. **a**, Cohort of patients originally suspected for *POMC* deficiency (*n* = 20) from the University of Berlin and the Leipzig Childhood Obesity cohort were analyzed for copy numbers of the genomic *ITCH*–*ASIP* fusion sequence normalized to the copy numbers of β-actin. Patients A–E were identified with *ASIP* mutations. Samples from the index patient (patient A, red) were used as a positive control. A patient not harboring the *ASIP* mutation served as control. Data are given as fold change compared to patient A. **b**, *ASIP* gene expression in PBLs of patients normalized to β-actin and TATA-box-binding protein. Data are given as fold change to the control patients (consisting of the twin of patient E, the mother of patient A and three patients of the Leipzig Childhood Obesity cohort). **c**, Comparison of BMI SDS and height SDS of patients with *ASIP* mutation (as indicated) with healthy population (*n* = 1,868; male *n* = 908; mean age 10.0 years; open black) and with Leipzig Obesity Childhood cohort (*n* = 1,734; male *n* = 861; mean age 11.6 years; open red) shown as violin plots with median and quartiles indicated as dashed and dotted lines, respectively. Patients with *ASIP* mutation have significantly higher height SDS (*P* = 0.0419 on two-sided Mann–Whitney *U*-test). Source data

We screened a targeted 20 patients with a phenotype indicative of defects in the POMC pathway (extreme obesity and red hair color) for *ASIP* variants originally suspected but not diagnosed for *POMC* deficiency. However, we did not identify a patient with a similar *ITCH*–*ASIP* fusion (Fig. 6a).

Targeted screening of our Leipzig Childhood Obesity cohort (*n* = 1,745) revealed four additional patients (three girls and one boy) carrying exactly the same mutation with the identical 5′*ITCH*–*ASIP* fusion as our index patient (Table 1). For three of them, RNA from peripheral blood was available and ectopic *ASIP* expression was confirmed (Fig. 6b). All four patients suffered from severe childhood obesity (Table 1). Compared to the obesity cohort, most patients carrying the *ASIP* mutation have a BMI SDS and height SDS above the median (Fig. 6c). Notably, one of these patients has a twin brother, who does not harbor the mutation. Even though obesity is present in both brothers, they are discordant in the extent of obesity (>25 kg difference in body weight, difference BMI SDS 0.67; height difference of 8.8 cm, difference height SDS 1.18; all at the age of 11 years; Table 1).

**Table 1:** Phenotype of patients carrying *ASIP* mutation

Patient	A (index)	B	C	D	E	Fraternal twin of E
**Carrier of** ***ASIP*** **mutation**	Yes	Yes	Yes	Yes	Yes	No
***ASIP*** **overexpression in PBLs**	Yes	Yes	Yes	Not available	Yes	No
**Sex**	Female	Female	Female	Female	Male	Male
**Age (years)**	12.37	3.78	9.33	9.95	11.36	11.36
**Pubertal stage**^**a**^	PH4, B4	PH1, B1	PH3, B2	PH1, B1	PH2, G1	PH2, G2
**Weight (kg)**	156.0	25.0	60.9	60.0	89.6	62.75
**Height (cm)**	181.0	109.5	143.9	149.8	160.7	151.9
**BMI SDS**	3.75	2.65	2.83	2.40	2.89	2.22
**Height SDS**	3.36	1.74	0.97	1.30	1.68	0.5
**Hair color**	Red	Red	Reddish	Reddish brown	Brown	Brown
**Hyperphagia**	Moderate	Severe	Moderate	Moderate	None	None
**Onset of overweight (obesity)**	1st YoL (1st YoL)	2nd YoL (2nd YoL)	2nd YoL (3rd YoL)	1st YoL (2nd YoL)	1st YoL (5th YoL)	1st YoL (5th YoL)
**Comorbidities**	Hyperinsulinemia, impaired glucose tolerance, hepatic steatosis, hyperuricemia	Hyperuricemia	Hyperinsulinemia, impaired glucose tolerance	Hyperinsulinemia, dyslipidemia	Bronchial asthma, dyslipidemia, global developmental delay	Global developmental delay

^a^Pubertal stages indicated by B, breast stage according to Tanner; G, genital stage (male) according to Tanner; and PH, pubic hair stage according to Tanner. YoL, year of life

To further support the link between the identified tandem duplication and childhood obesity, we screened healthy populational childhood cohorts (*n* = 2,051). We performed power calculation assuming that the prevalence in the general pediatric population were identical to the one we observed in our obesity group (0.28%). According to this, with a power of 80% (significance level 0.05), screening of 2,154 probands should be sufficient to identify six carriers of the *ASIP* mutation (corresponding to 1 in 359). We did not find any in the healthy population cohort of 2,051 probands that we screened (*P* = 0.02 with both Fisher’s exact test and a chi-squared test with Monte Carlo simulation), further supporting that the tandem duplication is indeed associated with obesity.

Hence, identification of the additional, unrelated patients even in our local sample holds promise of detecting more patients with ubiquitous *ASIP* expression if genetic diagnostic algorithms are extended to chromosomal rearrangements.

## Discussion

**Fig. 7:**
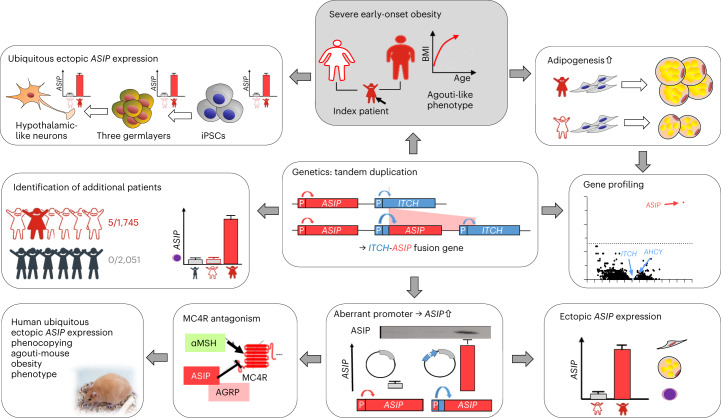
Graphical summary. Starting out from a case of a patient with severe early-onset obesity, accelerated linear growth, red hair and hyperinsulinemia (gray shaded box), we found enhanced adipogenesis of her SVF adipose-derived cells. Transcriptomic profiling identified aberrant *ASIP* expression, which is caused by chromosomal rearrangement with tandem duplication placing *ASIP* under the control of the *ITCH* promoter found in genetic profiling. Functionally verified aberrant promoter usage subsequently leads to ectopic *ASIP* expression as verified in native and patient-derived iPSCs of the index patient. ASIP antagonizes MC4R signaling and thereby ubiquitous ectopic *ASIP* expression can induce a phenotype phenocopying the agouti mouse models. Cohort screening identified four additional patients carrying the same *ASIP* mutation, whereas none was identified in a populational cohort. Carriers of the *ASIP* mutation are shown as red-shaded symbols, controls with obesity as white-shaded symbols and healthy children as gray symbols.

We report an agouti-like human monogenic obesity trait that presents with extreme early-onset obesity, accelerated linear growth beyond what would be expected for children with obesity^15^ and hypopigmentation. Based on experimental data from the analyses of patient-derived native and iPS cells, we identified ubiquitous ectopic *ASIP* expression due to chromosomal rearrangement as the likely cause of the phenotype (Fig. 7).

The identified tandem duplication leads to the generation of an ectopically expressed *ITCH*–*ASIP* fusion messenger RNA under control of the naturally ubiquitously active *ITCH* promoter and results in increased levels of secreted ASIP protein. In line with this, we found ectopic *ASIP* expression in several cell types of the patient (for example adipocytes and peripheral blood leukocytes). Even though desirable, we are not able to show ectopic *ASIP* expression directly in functional hypothalamic/brain circuits as we naturally do not have access to these patient tissues; however, patient-derived iPSCs differentiated into cells of all germ layers and further on into hypothalamic-like neurons, maintaining ectopic *ASIP* expression. These findings together with the fact that *ITCH* expression was detected in the brain and hypothalamus and that ASIP acts as an antagonist at the MC1R and MC4R, support that *ASIP* is ectopically expressed in the hypothalamus of the patient.

The murine *ASIP* homolog nonagouti is well known to be linked to obesity in rodents. The coding region of the human *ASIP* gene is 85% homologous to the mouse gene^16^ and there is high functional conservation^17^. Concordant with our patient, several rodent models with ubiquitous ectopic expression of nonagouti develop increased obesity, body length, yellow fur and hyperinsulinemia^9,11,12^. Notably, similar to that in naturally occurring agouti mice^9^, ectopic *ASIP* expression in our patient is caused by a gene rearrangement resulting in a change in promoter usage and hence ubiquitous and ectopic *ASIP* expression. Of note, there is a transgenic agouti mouse model, in which expression of the murine *ASIP* homolog is driven by the ubiquitously active human β-actin promoter, hence a very similar genetic constellation as we identified in our patient. These mice show overexpression of *ASIP* in the brain, leading to an obese phenotype with hyperphagia and hyperinsulinemia^10^.

**Extended Data Fig. 2:**
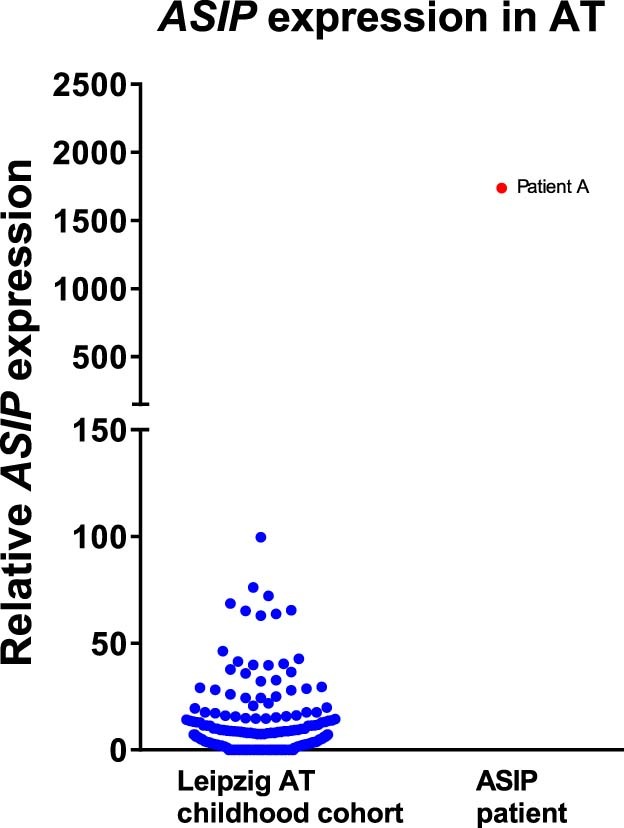
*ASIP* expression in adipose tissue samples of children. Gene expression levels of agouti signaling protein (*ASIP*) in subcutaneous adipose tissue (AT) of 328 children included in the Leipzig AT childhood cohort are shown in comparison to patient A. Data points are indicated for each subject. Gene expression data were normalized to the expression of β-actin, TATA-box-binding protein and hypoxanthine-guanine-phosphoribosyltransferase. Data points from controls are shown in blue and from the patient in red. Source data

Physiologically, the *ASIP* gene is expressed in hair follicles and antagonizes MC1R function on melanocytes and its ectopic expression, hence, causes yellow fur in mice. Although the physiological function of human ASIP does not seem to be entirely clear, we and others have shown it to be pharmacologically active and to antagonize the human melanocortin receptors^17^. Furthermore, genome-wide association studies revealed associations of variants at the *ASIP* locus with skin color, melanoma risk and red hair color, pointing toward a physiological function in hair and skin pigmentation similar to other mammals. Nevertheless, we did not find variants in *ASIP* or *MC1R* associated with red hair in our index patient, thus indicating that the pigmentation phenotype derives from the functional overexpression of *ASIP*. Apart from this, a study by Voisey et al. indicated that *ASIP* mRNA levels in adipocytes were sex-specifically correlated with BMI in adult human probands with opposite directions in men and women^18^. In contrast to this we found no or only spurious *ASIP* expression in subcutaneous adipose tissue samples of children from our Leipzig Adipose Tissue Childhood cohort^19^ (Extended Data Fig. 2).

The obesity phenotype is explained by murine nonagouti/ASIP and human ASIP sharing structural similarity with agouti-related peptide^20^. When ectopically expressed in the hypothalamus, ASIP is hypothesized to interfere with the leptin–melanocortin axis by competitively antagonizing the natural ligand α-melanocyte-stimulating hormone at the MC4R and thereby centrally affecting food intake and energy expenditure; however, ubiquitous expression of *ASIP* in mice has been reported to result in rather moderate hyperphagia compared to leptin- or leptin-receptor-deficient mice^11^. Similarly, our patient reported struggling with satiety before bariatric surgery and showed restrained eating, indicating a conscious counteracting of the hyperphagic drives that she sensed. Increased levels of incretins after bariatric surgery may have affected neuronal MC4R signaling in the patient, further facilitating restrained eating^21^. Besides hyperphagia, other mechanisms may affect the development of obesity^11,22^. Agouti-related peptide in the hypothalamus affects energy expenditure^23^, locomotor activity^24^ and sympathetic tone as well as shifting substrate utilization^25^ in peripheral tissues. Ectopic ASIP may have analogous effects. Indeed, we observed reduced spontaneous physical activity in our patient and observed a decreased resting metabolic rate at the whole body and cellular level, which might at least partially contribute to the obesity phenotype. The father suffers from obesity, diabetes and has red hair. In childhood and adolescence he engaged intensely in competitive sports, which affects energy balance beneficially, but did not entirely prevent obesity. Variable phenotype–genotype concordance is also known in other monogenic causes of obesity, for example *MC4R* mutations^26^. It has been described that *MC4R* mutations and variants exhibit partial penetrance and age-dependent expressivity^27^. Of note, our index patient reported that her hair changed with age, from deep red (as a toddler) to more reddish and brown (now as a young adult), which might indicate similar altered expressivity of the genotype–phenotype association. Although ectopic ASIP most likely promotes obesity primarily via central effects, ectopic ASIP may also contribute to obesity through peripheral effects as described for enhanced insulin secretion in the pancreas, leptin secretion from adipocytes, reduced lipolytic activity in adipocytes or regulation of adipogenic transcription factors^22,28^ and it may even affect activation of brown AT^29^. In line with this, the patient showed hyperinsulinemia and her SVF cells differentiated better into adipocytes than SVF cells from controls, indicating additional peripheral obesity-related effects of ubiquitous *ASIP* expression. Potentially, primary hyperinsulinemia may also drive the linear overgrowth, as recently shown for epigenetically driven hyperinsulinemia causing gigantism in mice^30^.

Notably, although the patient experienced prominent weight regain after bariatric surgery, she showed sustained metabolic improvement. Apart from the described restrictive eating behavior of the patient and/or a potential sustained effect on incretin balance^21^, another potential explanation for this might be the young age of the patient at surgery, as it has been shown previously that the benefit in remission of diabetes and hypertension from bariatric surgery was higher in adolescents compared to older patients^31^.

Likely there are more patients with undiagnosed ectopic *ASIP* expression, as the locus around *ASIP* is susceptible to genetic rearrangements^32^. In fact, patients with CNVs have been described in publicly available databases (for example DECIPHER). These are mainly larger deletions of >1 Mbps. The CNV gain per se (with retained endogenous promoter linkage) is, however, not sufficient for ubiquitous and ectopic *ASIP* expression. This is fully dependent on the position of the breakpoints and rejoining points. The crucial consequence of the specific tandem duplication and the breakpoint/reinsertion point in our patient(s) is that one copy of *ASIP* is placed under control of the ubiquitous *ITCH* promoter and this results in ectopic *ASIP* expression.

Such gain-of-function mutations have been described in other species^9,33,34^ and similar duplications resulting in an *ITCH*–*ASIP* fusion gene have been observed in sheep^35^ and in quail^36^. The chromosomal rearrangement is, however, not readily detectable by routine sequencing approaches but requires techniques identifying CNVs. Only by targeted re-evaluation we were able to find four additional patients in a cohort of 1,745 children with overweight and obesity carrying the same genomic rearrangement as our index patient. That this is also associated with ectopic *ASIP* expression in blood cells further supports the finding of the tandem duplication causing the ectopic *ASIP* expression and the obese phenotype. In fact, the frequency of ~0.28% *ASIP* mutations in this pediatric obesity cohort may even reach the range of *MC4R* mutation prevalence, so far the most frequent monogenic obesity trait^37^. The screening of more patients to confirm this and to specify the phenotype of this monogenic trait caused by ectopic *ASIP* expression is pertinent, particularly as MCR agonists, such as setmelanotide as recently approved for deficiencies in components of the leptin–melanocortin signaling pathway^7^, provide a promising treatment option for these patients; however, concomitant stimulation of MC1R with setmelanotide causes skin pigmentation and may change melanoma risk^38^. Another treatment option might be glucagon-like peptide-1 receptor (GLP-1R) agonists as they have been shown to mediate weight loss in patients with *MC4R* mutations to a similar degree as in patients with polygenic obesity^39^; however, they would not target the underlying pathomechanisms at the MC4R. Agonists that are selective for MC4R or specifically target hypothalamic neurons would constitute a promising treatment option^2^.

In summary, we report that a genomic rearrangement leads to ubiquitous expression of *ASIP* in patients with severe early-onset obesity and overgrowth. This phenotype is concordant with mouse agouti obesity models ectopically expressing the homolog nonagouti/ASIP. Both, murine and human ASIP interfere with the melanocortin receptor signaling, which likely explains the obese phenotypes.

## Methods

### Oversight

The index patient and children of the control group were participants in the Leipzig Adipose Tissue Childhood^19^ (NCT02208141, https://clinicaltrials.gov/ct2/show/NCT02208141, first registered August 2014) and the Leipzig Childhood Obesity (NCT04491344, https://clinicaltrials.gov/ct2/show/NCT04491344, first registered July 2020) cohorts^15^. Children from the age of 12 years and their guardians provided informed consent. The studies were approved by the Ethics Committee of the University of Leipzig (265/08, 007/04).

### Clinical and eating behavior data, energy expenditure

Height and weight were measured using calibrated devices to the nearest 1 mm and 0.1 kg, respectively. BMI and height were referenced to sex and age according to current guidelines^40^ and were given as SDSs. Children were categorized into overweight (1.28 < BMI SDS ≤ 1.88) or obese (BMI SDS > 1.88) groups according to current national consensus guidelines^41^.

Blood parameters were measured by standard laboratory procedures in the Institute of Laboratory Medicine of the University of Leipzig.

Eating behavior was assessed by self-reports using validated questionnaires. The Eating Disorder Examination Questionnaire^42,43^ assesses eating disorder psychopathology with subscales restraint, eating, weight and shape concern and behavioral features (for example binge eating). Patient scores were compared to a reference population of 1,354 women <44 years of age^44^ and father’s scores to a reference population of 1,166 men aged 44–64 years (ref. ^44^). The Dutch Eating Behavior Questionnaire^45^ evaluates emotional eating, external eating and restraint using sum scores from Likert scales. Patient scores were compared to a reference population of 1,394 women between 14–94 years of age^45^.

Resting energy expenditure of the patient was examined at the age of 15 years following an overnight fast using an indirect calorimeter (Quark RMR, Cosmed Germany) according to the manufacturer’s protocol. Expected metabolic rate was calculated according to her age, sex, height and body mass.

### Isolation and cultivation of SVF cells from adipose tissue

Subcutaneous AT samples were obtained from children undergoing bariatric or elective orthopedic surgery (Supplementary Table 1). SVF cells and adipocytes were isolated and preserved for later RNA isolation as previously described^19,46^. The remaining SVF cells were filtered through a nylon mesh with 30-µm pore size. Erythrocytes were removed with erythrocyte lysis buffer (0.154 M NH_4_Cl (Sigma-Aldrich), 0.01 M KHCO_3_ (Merck) and 0.1 mM EDTA (Sigma-Aldrich)). SVF cells were frozen in liquid nitrogen in Dulbecco’s modified Eagle medium/Ham F-12 culture medium (DMEM/F-12) (Life Technologies) containing 10% fetal bovine serum (FBS) (Biochrom) and 10% dimethyl sulfoxide (DMSO) (Sigma-Aldrich). Cells were thawed and 24 h after seeding cells were washed three times with phosphate-buffered saline to select for adipocyte precursor cells via plastic adherence. Isolated SVF cells were cultivated in DMEM/Ham F-12 culture medium containing 10% FBS and 100 U penicillin and 0.1 mg ml^−1^ streptomycin (Sigma-Aldrich) at 37 °C and 5% CO_2_ and passaged every 3–4 d.

### Functional effects of *AHCY* overexpression

Human SGBS cells were kindly supplied by M. Wabitsch (University of Ulm) and cultured as previously described^13^.

pCMV plasmid transfections were performed with the Neon Transfection System 100 µl kit (Invitrogen). The electroporation protocol was optimized to pulse voltage 1,300 V, pulse width 20 ms, pulse number 2 and a cell density of 6 × 10^6^ cells ml^−1^. SGBS cells were transfected with 5 µg of pCMV-AHCY plasmid, for *AHCY* overexpression or the pCMV-Entry (both oriGene) as a control, per 1 × 10^6^ cells. After electroporation, 500,000 cells per well were seeded in a 12-well format for differentiation assay.

### Proliferation assay

For assessing proliferation, 500 SVF cells were seeded at day 49, 71 and 89 of cultivation per well in 96-well plates in five replicates and cultivated for 8 d. After 1 and 8 d, cell viability was assessed by WST-1 assay (Roche, Applied Bioscience) according to the manufacturer’s protocol. Afterwards, cells were fixed, stained with Hoechst 33342 (Sigma-Aldrich) and counted by microscope. Doubling time (days) between d1 and d8 was calculated as (duration (8 d) × log_10_(2)) / (log_10_(cell number per image d1) – log_10_(cell number per image d8))^47^.

### Differentiation assay

For assessing adipogenic capacity, in technical triplicates 30,000 SVF cells at day 34, 49, 71 and 89 of cultivation were seeded per well in a 48-well dish or transfected SGBS cells in a 12-well format were used. Cells were grown to confluence for 24 to 48 h and differentiated according to the Poietics human adipose-derived stem cell-adipogenesis protocol (Lonza) for 8 d (SVF cells) or 10 d (transfected SGBS cells).

Differentiated cells were fixed with Roti-Histofix 4% (Carl Roth), double-stained with 10 µg ml^−1^ Nile red (Sigma-Aldrich) and 40 µg ml^−1^ Hoechst 33342 (Sigma-Aldrich). The percentage of differentiated cells is given as mean from *n* = 3 technical replicates calculated as (number of counted differentiated cell per microscopic image) / (total number of cells per image). A cell was defined to be differentiated when containing at least two lipid droplets. Additionally, the cells were stained with Oil Red O (Sigma-Aldrich) solution (0.3% in 60% isopropanol) for 15 min and washed with H_2_O. Oil Red O was extracted with isopropanol and absorption was measured at 540 nm.

### Bioenergetic profiling

Mitochondrial function was assessed using the Seahorse XFe24 Analyzer (Agilent Technologies) and the XF Cell Mito Stress Test kit (Agilent Technologies) using concentrations of 2 µM oligomycin, 3 µM carbonyl cyanide-4 (trifluoromethoxy) phenylhydrazone and 1 µM rotenone/antimycin A. A total of 15,000 SVF cells from the patient or control children were seeded into gelatin-coated wells of XFe24-plates in 3–4 technical replicates. Measurements were performed 48 h post-transfection using XF Base Medium (Agilent Technologies) containing 2 mM pyruvate (Sigma-Aldrich), 10 mM glucose (Sigma-Aldrich) and 2 mM glutamine (Sigma-Aldrich). Results were normalized to total protein per well (µg) by lysing the cells in 30 µl 50 mM NaCl solution (Carl Roth) with subsequent protein quantification using the Pierce BCA Protein Assay kit (Thermo Fisher Scientific).

### Isolation of nucleic acids

RNA was isolated from SVF cells and reverse-transcribed as previously described^48^. From blood, RNA was extracted using the PAXgene Blood RNA Tubes protocol (PreAnalytics). RNA from different human tissues pooled from several individuals were obtained from Clontech-TakaraBio. Genomic DNA was extracted from EDTA blood samples using the QIAamp DNA Blood Mini kit (QIAGEN).

### Transcriptome-wide analyses

Gene expression was measured using a HumanHT-12 v4 BeadChip array (Illumina). The R package limma was used to perform preprocessing (that is, background correction and quantile normalization) and differential gene expression analyses^49^ (10.5281/zenodo.7223530). Fold changes were log_2_-transformed and *P* values were adjusted for multiple testing using the Benjamini–Hochberg procedure (FDR).

### qRT–PCR

Quantitative real-time PCR (qRT–PCR) was performed as previously described^48^ using the QuantStudio3 Real-Time PCR System (Applied Biosystems) with the qPCR Master Mix Plus - Low ROX (Eurogentec) or the Takyon Low ROX Probe Master Mix dTTP Blue (Eurogentec). Gene expression was quantified using a standard curve of serial dilutions from a linearized plasmid containing the target sequence or from a reference sample. Gene expressions were normalized to the expression of β-actin and TATA-box-binding protein. For measurement of *ASIP* expression in subcutaneous adipose tissue samples from the Leipzig Adipose Tissue Childhood cohort hypoxanthine-guanine-phosphoribosyltransferase was used as a third reference gene. Supplementary Appendix and Supplementary Table 4 lists all sequences of primers and probes and TaqMan Gene Expression Assays (Thermo Fisher Scientific) used for qRT–PCR.

### 5′-RACE–PCR

5′-RACE–PCR was performed from peripheral blood RNA using the SMARTer RACE cDNA Amplification kit (Clontech Laboratories) using primers in Supplementary Appendix and Supplementary Table 5.

### RNA-seq and transcript analyses

RNA was isolated from SVF cells of the patient and control children 1–4 as well as from peripheral blood mononuclear cells of the patient and control 9 using TRI REAGENT (Sigma-Aldrich). RNA quantity was measured with a spectrometer (Nanodrop ND 1000). We included only RNA samples with RIN values >8 analyzed on the Agilent 2100 bioanalyzer using the RNA 6000 Nano Chip (Agilent Technologies). Indexed cDNA libraries were generated using TruSeq RNA Sample Preparation kits v2 (Illumina). The average library size was 300 bp as determined on the Agilent 2100 Bioanalyzer with DNA 1000 Chips. The libraries were sequenced on the Illumina HiScanSQ Sequencing System (Macrogen).

Reads were mapped to the reference human genome (GRCh38.p13, INSDC Assembly GCA_000001405.28, Dec 2013) using TopHat (v.2.1.1)^50^. After indexing with samtools (v.1.9)^51^ the mapped reads were assembled to transcripts and quantified by StringTie (v.2.1.3b)^52,53^. For TopHat, we used the ‘default’ parameters. StringTie parameters ‘read coverage’ (–c), ‘transcript length’ (–m) and ‘bases on both sides of a junction a spliced read has to cover’ (–a) were set to minimal values. The parameter ‘fraction of most abundant transcript at one locus’ (–f) was lowered from default (0.01) to 0 as correction for artifacts and incompletely processed mRNA with a 1% cutoff was performed after the comparative analysis. For all other StringTie parameters default values were used. Assembled transcripts were inspected with the Integrated Genome Viewer (Broad Institute)^54,55^ and samples showing a visible 3′ bias due to oligonucleotide-dT/poly-A primer selection were not included.

Due to small sizes of the exons of *ITCH* and *ASIP* coverage, analyses of single exons were not possible. Instead, a de novo assembly of *ITCH* and *ASIP* transcripts was performed using StringTie. Three new transcript variants occurred containing only the first two annotated exons of *ITCH*. The fragments per kilo base per million mapped reads for these variants and also of the *ASIP* and *AHCY* transcripts were compared between the patient and the controls.

### Genomic and genetic analyses

Trio WGS and bioinformatic analyses were performed at the DRESDEN-concept Genome Center. After ultrasonic shearing of 800 ng gDNA (LE220, Covaris) the DNA library preparation was performed using the Kapa HyperPlus kit (Roche) according the manufacturer’s instructions. After ligation with uniquely dual indexed adaptors (IDT), non-ligated adaptors were removed by adding XP beads (Beckmann Coulter) in a ratio of 1:0.9. The DNA libraries were then size selected with XP beads to an average insert size of 300 bp and quantified by qPCR (LightCycler 480, Roche) and the Fragment Analyzer (Agilent). Libraries were sequenced paired end 2 × 150 bp to a coverage >38× on a NovaSeq 6000 (Illumina). Bioinformatic analysis was performed using the Illumina DRAGEN pipeline (07.021.595.3.7.5).

The TruSight One Sequencing panel was performed after enrichment with Nextera DNA Flex Pre-Enrichment LibraryPrep and Enrichment, IDT for Illumina Nextera UD Indexes and the NextSeq 500/550 High Output v2 kit (300 cycles) using the Illumina NextSeq 500/550. Data were analyzed using the software Varvis and Varfeed (Limbus). All analyses were performed according to American College of Medical Genetics and Genomics criteria.

Molecular karyotyping of genomic DNA was performed by array comparative genomic hybridization using the Infinium CytoSNP-850K v.1.2 BeadChip (Illumina) and analyzed using BlueFuse Multi (v.4.4).

### Luciferase reporter and expression assays

DNA sequences were cloned according to the TOPO TA Cloning kit, Dual Promoter protocol and confirmed by sequencing by the Core Unit for DNA technologies of the University of Leipzig. Primers used for cloning are indicated in Supplementary Appendix and Supplementary Table 5.

For luciferase assays the *ITCH* promoter was inserted upstream the luciferase reporter gene into the pGL3-Basic vector using the restriction enzymes *Sac*I and *Xho*I.

For generation of modified pGL3-Basic expression vectors containing the *ASIP* or the *ITCH*–*ASIP* coding sequence under control of *ITCH* or no promoter, the luciferase reporter gene was cut out of the pGL3-Basic vector using *Nco*I and *Xba*I and the cloned mRNA sequences were inserted. Subsequently, the promoter sequence of *ITCH* was inserted into those vectors using the restriction enzymes *Sac*I and *Xho*I.

A second independent expression vector containing the coding sequence of *ASIP* fused to the 5′ UTR of *ITCH* under control of the *ITCH* promoter (2.8 kbp upstream of transcription start) was generated by Taconic Bioscience, as shown in Supplementary Appendix and Supplementary Fig. 1.

For luciferase reporter assays, 500,000 human embryo kidney cell line 293 (HEK293) (Sigma-Aldrich, ECACC 85120602) cells were seeded per well (six-well dish plate) and were co-transfected 24 h post-seeding with 1 µg of pGL3-Basic plasmids containing the firefly luciferase reporter under control of a 2,868 bp *ITCH* promoter sequence or no promoter and 50 ng of pRL-CMV control plasmid containing the luciferase gene from *Renilla* *reniformis* using 3 µl Fugene HD transfection reagent (Promega). Luciferase activities were measured using the Dual-Luciferase Reporter Assay System (Promega) according to the manufacturer’s protocol 48 h post-transfection using a CLARIOstar plate reader (BMG Labtech). Experiments were performed in technical duplicates. For normalization, the sum of firefly luciferase signal was divided by the sum of *Renilla* luciferase signal.

### Generation and differentiation of patient-derived iPSCs

The generation of iPSCs from SVF cells isolated from adipose tissue of the patient and two control patients, 1 and 10, was performed by the iPSC Core Facility, Institute of Stem Cell Research, Helmholtz Zentrum Munchen. Pluripotency was confirmed by staining of OCT4, SOX2, LIN28 and NANOG^56^. The iPSCs were cultivated on Geltrex (Thermo Fisher Scientific)-coated cell culture plates in mTeSR 1 medium (Stem Cell Technologies) at 37 °C and 5% CO_2_.

Characterization of the pluripotency of patient-derived iPSCs was performed as monolayers by directed differentiation into the ectodermal^57^, mesodermal^58^ and endodermal^59^ germ layers for a period of 5 d and subsequent qRT–PCR analyses of marker genes for each of the germ layers, as previously described^56^. Relative expression levels were calculated using the delta-delta Ct method normalized to β-actin.

Differentiation of patient-obtained iPSCs and the HMGU1 control iPSC line (kindly supplied by iPSC Core Facility, Institute of Stem Cell Research, Helmholtz Zentrum Munchen) into HTNs followed the previously described protocol of Wang et al.^60^. For generating hypothalamic NPCs, 500,000 iPSCs per well were cultured on Geltrex-coated 12-well-plates as described in the protocol and collected at day 12. For further differentiation, 300,000 single cells per well were plated on poly-l-ornithine- (0.01%, Sigma-Aldrich) and laminin-coated (4 µg ml^−1^; Thermo Fisher Scientific) 24-well plates. After cells had attached to the plate, the medium was replaced with N2 medium with B27 and 10 μM DAPT. Four days later, the medium was changed to N2 medium with B27 and 20 ng ml^−1^ BDNF (R&D) and cells were cultured until day 26.

### Immunoblotting

For analysis of ASIP protein secretion 800,000 SVF cells were seeded on 15-cm plates, cells and supernatants were collected after 24 h of incubation with the secretory pathways inhibitors 5 µg ml^−1^ brefeldin A (in DMSO; BioLegend) and 2 µM monensin (in 70% ethanol; BioLegend) or the control medium containing the same volume of DMSO and ethanol.

For analysis of ASIP protein generation from the fusion construct, 600,000 HEK cells were seeded per six-well dish and transfected 24 h later with 2 µg plasmid using 8 µl using Fugene HD transfection reagent (Promega) per well. Medium was changed 24 h after transfection. Cells and supernatants were collected 48 h after medium change for immunoblotting.

For the analysis of ASIP protein generation and secretion in the iPSC lines, cells were grown in six-well-plates to confluence before the medium was changed to 1 ml knockout DMEM with 1× MEM NEAA, 1× β-mercaptoethanol, 0.1% BSA (Serva), 100 U penicillin, 0.1 mg ml^−1^ streptomycin and 2 mM glutamine. After 24 h, cells and supernatants were collected for immunoblotting.

Conditioned medium was centrifuged for 5 min at 800*g* and the supernatant lyophilized and re-suspended in H_2_O for receiving 10x concentrated medium. Cells were lysed in RIPA lysis buffer (50 mM Tris pH 7.5, 150 mM NaCl, 1% Triton X-100 and 0.1% SDS, with cOmplete Mini Protease Inhibitor Cocktail Tablets; Roche, Sigma-Aldrich) with additional break-up via QiaShredder Homogenizer Columns (QIAGEN). The protein concentration of cell lysates was measured using the Pierce BCA Protein Assay kit (Thermo Fisher Scientific). Equal amounts of protein or medium were resolved by 12% or 15% SDS–PAGE and ASIP was detected using an ASIP antibody (PA5-77052, Invitrogen, 1:1,000 dilution). Equal loading was confirmed by detection of β-actin using a β-actin antibody (ab8227, Abcam, 1:1,000 dilution).

### Melanocortin receptor signaling by cAMP assay

A Chinese hamster ovary cell line CHO-K1 (ATCC CCL-61) was grown in DMEM/F-12 supplemented with 10% FBS, 100 U ml^−1^ penicillin and 100 µg ml^−1^ streptomycin. Cells were at seeded at 0.9 × 10^6^ cells per 25-cm^2^ flask and transfected with plasmid (3 µg total amount of DNA) and Lipofectamine 2000 (Thermo Fisher Scientific). Plasmids encoding human MC1R^61^, human MC4R^62^ and empty vector pcDps have previously been described.

One day after transfection, cells were split into 96-well plates (2 × 10^4^ cells per well) and serum-starved for 16 h before the experiment. cAMP content of cell extracts was determined by a non-radioactive assay with ALPHAScreen (PerkinElmer) technology as previously described^63^. In brief, stimulation with various α-MSH (Sigma-Aldrich) concentrations in the absence or presence of 100 nM ASIP (R&D systems) or with various concentrations of ASIP alone were performed 48 h after transfection. Reactions were stopped by aspiration of medium and cells were lysed in 20 μl of lysis buffer containing 1 mM 3-isobutyl-1-methylxanthine. From each well, 5 μl of lysate were transferred to a 384-well plate. Acceptor beads and donor beads were added according to the manufacturers’ protocol. Cyclic AMP accumulation data were analyzed using GraphPad Prism v.8.4.3.

### Screening for patients with *ASIP* mutations

In unselected cohorts of *n* = 1,745 children of the Leipzig Childhood Obesity cohort (mean age, 11.6 years; female, *n* = 879; male, *n* = 867) as well as 2,051 children of healthy population childhood cohorts (Leipzig childhood cohort, *n* = 1,447; mean age, 11.7 years; female, *n* = 755; male, *n* = 692; Leipzig Lifestyle and environmental factors and their Influence on Newborns Allergy risk (LINA) cohort^64^, *n* = 604; mean age, 4 years; female, *n* = 291; male, *n* = 313) we screened for carriers of the genomic *ITCH*–*ASIP* fusion. The studies were approved by the ethics committee of the University of Leipzig (Leipzig childhood cohort, reg. nos. 007-04/027-04, 782-1998, and 029-2006; LINA cohort, reg. nos. 046-2006 and 144-10-31052010, registered in https://www.birthcohorts.net since 6 May 2019). Children from the age of 12 years on and their guardians provided informed consent. Twenty patients with obesity and red hair color, who were previously suspected for mutations in the *POMC* gene were additionally screened for genomic *ITCH*–*ASIP* fusion. These patients were recruited at the Freie Universitat Berlin, Humboldt-Universitat zu Berlin, Institute for Experimental Pediatric Endocrinology (mean age, 9.4 years; female, *n* = 13; male, *n* = 7). This analysis was approved by the local ethical committee (EA2/131/11).

gDNA copy numbers of the *ITCH*–*ASIP* fusion sequence were quantified using SYBR green qRT–PCR using the Maxima SYBR Green/ROX qPCR Master Mix (2×) (Thermo Fisher Scientific) and the *ITCH*–*ASIP* fusion primers (Supplementary Appendix and Supplementary Table 5) with an input of 2–10 ng gDNA per reaction. Copy numbers were normalized to copy numbers of β-actin. On each plate, samples from the patient were carried along as a positive control.

For comparison of BMI SDS and height SDS of identified carriers with *ASIP* mutation with the obesity cohort, we excluded eight patients with underlying syndromic or endocrine diseases (Prader–Willi syndrome, growth hormone deficiency, syndromic short stature or autoimmune polyendocrinopathy) and a further three patients had missing data for either height or BMI. Of the LINA birth cohort, from *n* = 183 probands, data and samples were only available at birth and were, therefore, excluded from comparative anthropometric analyses.

### Statistical analyses

If not otherwise stated, statistical analyses were performed using a Student’s *t*-test (two-sided) in GraphPad Prism 6 (GraphPad Software). *P* values <0.05 were considered statistically significant.

### Reporting summary

Further information on research design is available in the Nature Portfolio Reporting Summary linked to this article.

## Supplementary information

Supplementary Information Supplementary Tables 1–5, Supplementary Fig. 1 and Supplementary References.

Reporting Summary

## Data Availability

The datasets generated during and/or analyzed during the current study are deposited as source files except for patient individualized datasets that underly patient confidentiality restrictions but are available from the corresponding author upon reasonable request. Source data are provided with this paper.

## Source data

Source Data Fig. 1 Statistical Source Data.
Source Data Fig. 2 Statistical Source Data.
Source Data Fig. 3 Statistical Source Data.
Source Data Fig. 3 Unprocessed and uncropped images of western blots; RAW images Fig. 3.
Source Data Fig. 4 Statistical Source Data.
Source Data Fig. 4 Unprocessed and uncropped images of agarose gel and western blots; RAW images Fig. 4.
Source Data Fig. 5 Statistical Source Data.
Source Data Fig. 5 Unprocessed and uncropped images of western blots; RAW images Fig. 5.
Source Data Fig. 6 Statistical Source Data.
Source Data Extended Data Fig. 1 Statistical Source Data.
Source Data Extended Data Fig. 2 Statistical Source Data.
