# Supplementary material for: Aberrant expression of agouti signaling protein (ASIP) as a cause of monogenic severe childhood obesity
Source: Nat Metab. 2022 Dec 19;4(12):1697–712. doi: 10.1038/s42255-022-00703-9 (PMC9771800; doi:10.1038/s42255-022-00703-9)
Supplement: Supplementary file 1 — Supplementary Tables 1–5, Supplementary Fig. 1 and Supplementary References. [file 42255_2022_703_MOESM1_ESM.pdf]

nature metabolism

Article

https://doi.org/10.1038/s42255-022-00703-9

Aberrant expression of agouti signaling protein (*ASIP*) as a cause of monogenic severe childhood obesity

In the format provided by the authors and unedited

Supplementary Information

1. Supplementary Tables

Table S1. Clinical characteristics of the index patient and control children with adipose tissue samples

**available**	**Adipose tissue samples**						**Gene expression data from PBL**					**PBL RNA sequencing**	
	**Patient**	**Ctr 1**	**Ctr 2**	**Ctr 3**	**Ctr 4**	**Ctr 10**	**Patient**	**Ctr 5**	**Ctr 6**	**Ctr 7**	**Ctr 8**	**Patient**	**Ctr 9**
**Anthropometric data**													
Age (years)	12.4	17.4	13.3	11.8	12.7	14.7	14.1	16	12.8	12.3	15.0	15.0	46.2
Sex	female	female	male	female	female	male	female	female	female	female	female	female	female
Height (cm) / Height SDS	181 / 3.4	176 / 1.3	156 / 0.0	158 / 0.7	160 / 0.3	163 / -1.0	183 / 2.9	167 / 0.1	186 / 3.8	163 / 1.0	183 / 2.7	183 / 2.8	173
BMI (kg/m²) / BMI SDS	47.6 / 3.7	40.5 / 3.7	25.5 / 1.8	31.4 / 2.7	29.7 / 2.4	22.2 / 0.8	37.8 / 3.2	33.1 / 2.8	44.7 / 3.6	31.0 / 2.6	38.6 / 3.3	39.7 / 3.4	19.0
Surgery	sleeve gastrectomy	sleeve gastrectomy	removal 8P	implan-tation 8P	implan-tation 8P	implan-tation 8P	---	---	---	---	---	---	---
Location	abdomen	abdomen	femur	knee	knee	knee	---	---	---	---	---	---	---

Ctr, control; SDS, standard deviation score; BMI, body mass index; 8P, eight plate; PBL, peripheral blood leukocytes

Table S2. RNA sequencing results from SVF cells from the patient and control children

Subject	Patient	Patient	Ctr 1	Ctr 2	Ctr 3	Ctr 4
**SVF cell passage (p)**	**p15**	**p28**	**p16**	**p19**	**p19**	**p14**
ASIP (FPKM)	5.07	4.89	0.20	0.36	0.18	0.22
AHCY (FPKM)	14.17	11.94	9.25	7.93	12.74	10.19
ITCH (FPKM)	19.24	25.77	21.31	18.67	21.92	24.46

SVF, stromal vascular fraction; Ctr, control; SDS, standard deviation score; FPKM, fragments per kilobase of transcript per million fragments mapped. *ASIP*, agouti signaling protein; *AHCY*, adenosylhomocysteinase; *ITCH*, itchy E3 ubiquitin protein ligase

Table S3. Variants strongly associated with red hair according to Morgan *et al.*[1] in the patient and her father

Gene	Variant ID	Effect	Associated with red hair	Patient	Father
MC1R	rs1805006	D84E	A	C/C	C/C
MC1R	rs1805007	R151C	T	C/C	C/C
MC1R	rs1110400	I155T	C	T/T	T/T
MC1R	rs1805009	D294H	C	G/G	G/G
MC1R	rs1805008	rg160Trp or R160W	T	C/C	C/C
MC1R	rs34357723	97kb from the 5' end of MC1R	T	C/C	C/C
MC1R	rs368507952	R306H	A	G/G	G/G
MC1R	rs200000734	R213W	T	C/C	C/C
MC1R	rs201326893	Y152X	A	C/C	C/C
MC1R	rs11547464	R142H	A	G/G	G/G
MC1R	rs3212379	upstream	T	C/C	C/C
MC1R	rs34474212	S83P	C	T/T	T/T
MC1R	rs34158934	T95M	T	C/C	C/C
MC1R	rs555179612	I182Hfs	TC	C/C	C/C
RALY/ASIP	rs6059655	non-coding	A	G/G	G/G

The affected gene, variant ID, effect and the allele associated with red hair is shown, together with the genotypes of the patient and her father. *MC1R*, melanocortin receptor 1; *RALY*, RALY heterogeneous nuclear ribonucleoprotein; *ASIP*, agouti signaling protein

Table S4. Primer sequences for qPCR

Target	Primer, forward	Primer, reverse	Probe/SYBR green
**Gene expression**			
ACTB	CGAGCGCGGCTACAGCTT	CCTTAATGTCACGCACGATTT	ACCACCACGGCCGAGCGG
ADIPOQ	GGC CGT GAT GGC AGA GAT	CCT TCA GCC CCG GGT ACT	CGATGTCTCCCTTAGGACCAATAAGACCTGG
AGRP	GGATCTGTTGCAGGAGGCTCAG	TGAAGAAGCGGCAGTAGCACGT	GCCCGCTCCTCACGTCGCT
AHCY	Taqman Assay Hs04183463_g1(4331182, Thermo Fisher Scientific Inc.		
AP2	GCTTTTGTAGGTACCTGGAAACTTG	ACACTGATGATCATGTTAGGTTTGG	CCTGGTGGCAAAGCCCACTCCTCAT
ASIP	CAACTCCTCTGTGAACCTACTGGAT	TGCCGATCTGTTTGGATTTCT	CCCTTCTGTCTCTATTGTGGCGCTGAAC
CXCR4	CACCGCATCTGGAGAACCA	GCCCATTTCCTCGGTGTAGTT	SYBR green
FOXA2	GGGAGCGGTGAAGATGGA	TCATGTTGCTCACGGAGGAGTA	SYBR green
HPRT	GGCAGTATAATCCAAAGATGGTCAA	GTCTGGCTTATATCCAACACTTCGT	CAAGCTTGCTGGTGAAAAGGACCCC
ITCH	TaqMan Assay Hs00395201_m1 (4331182, Thermo Fisher Scientific Inc)		
LEPR	TTGTGCCAGTAATTATTTCCTCTT	CTGATCAGCGTGGCGTATTT	SYBR green
MC4R	CTTATGATGATCCCAACCCG	GTAGCTCCTTGCTTGCATCC	TGCTTTCAATTGCAGTGGACAGGTACT
MESP	CTGCCTGAGGAGCCCAAGT	GCAGTCTGCCAAGGAACCA	SYBR green
MIXL	CCGAGTCCAGGATCCAGGTA	CTCTGACGCCGAGACTTGG	SYBR green
NANOG	CCTTCCTCCATGGATCTGCTT	CTTGACCGGGACCTTGTCTTC	SYBR green
Nestin	GTCTCAGGACAGTGCTGAGCCTTC	TCCCCTGAGGACCAGGAGTCTC	SYBR green
OCT4	CAATTTGCCAAGCTCCTGAAG	AAAGCGGCAGATGGTCGTT	SYBR green
PAX6	TCTTTGCTTGGGAAATCCG	CTGCCCGTTCAACATCCTTAG	SYBR green
POMC	CAGCCAGTGTCAGGACCTC	AAGTGGCCCATGACGTACTT	GGAGTGCATCCGGGCCTGCA
PPARG	GATCCAGTGGTTGCAGATTACAA	GAGGGAGTTGGAAGGCTCTTC	TGACCTGAAACTTCAAGAGTACCAAAGTGCAA
SOX1	GAGAACCCCAAGATGCACAA	CCTCGGACATGACCTTCCA	SYBR green
SOX2	CCTCCGGGACATGATCAGCATGTA	GCAGTGTGCCGTTAATGGCCGTG	SYBR green
SOX17	GGCGCAGCAGAATCCAGA	CCACGACTTGCCCAGCAT	SYBR green
T	CAACCTCACTGACGGTGAAAAA	ACAAATTCTGGTGTGCCAAAGTT	SYBR green
TBP	TTGTAAACTTGACCTAAAGACCATTGC	TTCGTGGCTCTCTTATCCTCATG	AACGCCGAATATAATCCCAAGCGGTTTG
**gDNA copy numbers**			
ACTB	CGAGCGCGGCTACAGCTT	CCTTAATGTCACGCACGATTT	SYBR green
ITCH-ASIP fusion	TGGAGAAAAGGAATCATCAGAGGA	GTGGTGAGCAAGGGACGTTTCAGC	SYBR green

Sequences are given in 5’ to 3’ direction. As dye/quencher FAM/TAMRA or HEX/TAMRA was used. If SYBR green was used this is indicated in the last column. *ACTB*, beta actin; *ADIPOQ*, adiponectin; *AGRP*, agouti-related protein; *AHCY*, adenosylhomocysteinase; *AP2*, adipocyte protein 2; *ASIP*, agouti signaling protein; *CXCR4*, C-X-C Motif Chemokine Receptor 4; *FOXA2*, Forkhead Box A2; *HPRT*, hypoxanthine-guanine-phosphoribosyltransferase; *ITCH*, itchy E3 ubiquitin protein ligase; *ITCH-ASIP* fusion, unique sequence of the *ITCH-ASIP* fusion gene; *LEPR*, leptin receptor; *MC4R*, melanocortin 4 receptor; *MESP*, Mesoderm Posterior BHLH Transcription Factor; *MIXL*, Mix Paired-Like Homeobox; *NANOG*, Nanog Homeobox; *OCT4*, POU Class 5 Homeobox 1; *PAX6*, Paired Box 6; *POMC*, proopiomelanocortin; *PPARG*, peroxisome proliferator-activated receptor gamma; *SOX1*, SRY-Box Transcription Factor 1; *SOX2*, SRY-Box Transcription Factor 2; *SOX17*, SRY-Box Transcription Factor 17; *T*, T-Box Transcription Factor T; *TBP*, TATA-box binding protein

Table S5. Primer sequences for 5' RACE PCR and cloning experiments

**5' RACE PCR**	***ASIP*-specific prime**	**nested *ASIP*-specific primer**
	TTGAGGCTGAGCACGCGGCAGGAGCAGG	CTGCGGAAGAAGCGGCACTGGCAGGAGG
**Cloning**	**Forward**	**Reverse**
ITCH-ASIP fusion gDNA	TGGAGAAAAGGAATCATCAGAGGA	GTGGTGAGCAAGGGACGTTTCAGC
**For Vector construction:**		
2,868 bp *ITCH* promoter sequence (NM031483.7)	CTGGAGCTCGAAGTGGTTTTGAAAGTACTTTGCT	ACTGCTCGAGAAAGCGCAGGCGCCTGAGCGCG.
ASIP mRNA sequence (NM001672.2)	ACTGCCATGGGCCTCCTGGGATGGATGTCACCC	ACTGTCTAGATGGGGGCGCTCAGCAGTTGAGGC
ITCH-ASIP mRNA	ACTGCCATGGAGCTGTGGTCGGGGCTCGGGAC	ACTGTCTAGATGGGGGCGCTCAGCAGTTGAGGC

Sequences are given in 5’ to 3’ direction. 5' RACE PCR, 5’ Rapid amplification of cDNA-ends and polymerase Chain Reaction; *ASIP*, agouti signaling protein; *ITCH*, itchy E3 ubiquitin protein ligase; *ITCH-ASIP* fusion, unique sequence of the *ITCH-ASIP* fusion gene.

2. Supplementary Figures

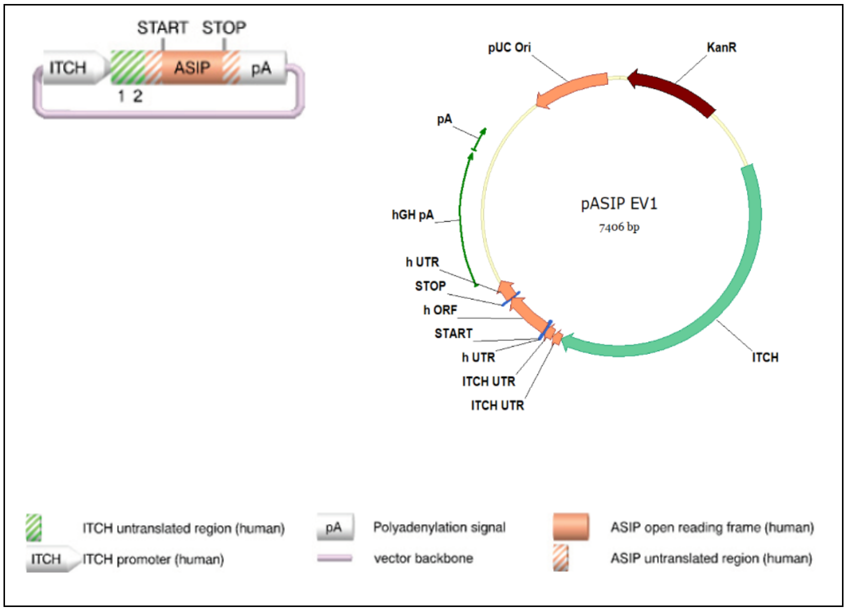

Figure S1. Taconic expression vector containing the *ITCH-ASIP* fusion mRNA sequence under control of the *ITCH* promoter

A scheme of the plasmid including the legend and the plasmid map of the construct is shown. *ITCH*, itchy E3 ubiquitin protein ligase; *ASIP*, agouti signaling protein

3. Supplementary References

1. Morgan MD, Pairo-Castineira E, Rawlik K, et al. Genome-wide study of hair colour in UK Biobank explains most of the SNP heritability. Nat Commun 2018;9:5271.
