# Supplementary material for: Aberrant expression of agouti signaling protein (ASIP) as a cause of monogenic severe childhood obesity
Source: Nat Metab. 2022 Dec 19;4(12):1697–712. doi: 10.1038/s42255-022-00703-9 (PMC9771800; doi:10.1038/s42255-022-00703-9)
Supplement: Supplementary file 2 — Reporting Summary [file 42255_2022_703_MOESM2_ESM.pdf]

nature research

Corresponding author(s): Antje Körner
Last updated by author(s): Mar 10, 2022

Reporting Summary

Nature Research wishes to improve the reproducibility of the work that we publish. This form provides structure for consistency and transparency in reporting. For further information on Nature Research policies, see our Editorial Policies and the Editorial Policy Checklist.

Statistics

For all statistical analyses, confirm that the following items are present in the figure legend, table legend, main text, or Methods section.

n/a	Confirmed	
☐	☒	The exact sample size (*n*) for each experimental group/condition, given as a discrete number and unit of measurement
☐	☒	A statement on whether measurements were taken from distinct samples or whether the same sample was measured repeatedly
☐	☒	The statistical test(s) used AND whether they are one- or two-sided<br>*Only common tests should be described solely by name; describe more complex techniques in the Methods section.*
☐	☒	A description of all covariates tested
☐	☒	A description of any assumptions or corrections, such as tests of normality and adjustment for multiple comparisons
☐	☒	A full description of the statistical parameters including central tendency (e.g. means) or other basic estimates (e.g. regression coefficient) AND variation (e.g. standard deviation) or associated estimates of uncertainty (e.g. confidence intervals)
☐	☒	For null hypothesis testing, the test statistic (e.g. *F*, *t*, *r*) with confidence intervals, effect sizes, degrees of freedom and *P* value noted<br>*Give P values as exact values whenever suitable.*
☒	☐	For Bayesian analysis, information on the choice of priors and Markov chain Monte Carlo settings
☒	☐	For hierarchical and complex designs, identification of the appropriate level for tests and full reporting of outcomes
☒	☐	Estimates of effect sizes (e.g. Cohen's *d*, Pearson's *r*), indicating how they were calculated

Our web collection on statistics for biologists contains articles on many of the points above.

Software and code

Policy information about availability of computer code

Data collection

Resting energy expenditure of the patient was measured using an indirect calorimeter (Quark RMR, Cosmed Germany).
Absorbance and fluorescence of cell were analyzed using the BZ-8000 microscope (Keyence, Neu-Isenburg, Germany).
Mitochondrial function was assessed using the Seahorse XFe24 Analyzer (Agilent Technologies).
Transcriptome-wide gene expression was measured using a HumanHT-12 v4 BeadChip array (ILLUMINA) by the Core Unit for DNA technologies of the University Hospital Leipzig.
TaqMan quantitative real-time polymerase chain reaction (qRT-PCR) was performed using the QuantStudio3 or QuantStudio6 Real-Time PCR System (Applied Biosystems®).
RNA quantity was measured with a spectrometer (Nanodrop ND 1000).
RNA quality was analyzed on the Agilent 2100 bioanalyzer using the RNA 6000 Nano Chip (Agilent Technologies, USA).
For whole genome sequencing the DNA libraries were quantified by qPCR (LightCycler 480, Roche) and the Fragment Analyzer (Agilent).
Libraries were sequenced paired end 2 x 150 bp to a coverage > 38x on a NovaSeq 6000 (ILLUMINA). cDNA libraries for RNA sequencing were on the Illumina HiScanSQ Sequencing System (Fa Macrogen Europe).
Luciferase activities were measured using a CLARIOstar plate reader (BMG Labtech).

Data analysis	If not otherwise stated, statistical analyses were performed using Student's t-test (two-sided) in GraphPad Prism 6 (GraphPad Software, San Diego, CA, version 6.07).<br>For RNA sequencing reads were mapped to the reference human genome (GRCh38.p13 (Genome Reference Consortium Human Build 38), INSDC Assembly GCA_000001405.28, Dec 2013) using Tophat. After indexing with samtools the mapped reads were assembled to transcripts and quantified by StringTie.<br>For whole genome sequencing bioinformatic analysis was performed using the ILLUMINA DRAGEN pipeline (07.021.595.3.7.5).<br>TruSight One Sequencing panel were analysed using the software Varvis and Varfeed CNV (Limbus, Rostock).<br>Molecular karyotyping of genomic DNA has been analysed using BlueFuse Multi (version 4.4).<br>Cyclic AMP accumulation data from the ALPHAScreen cAMP assay were analysed using GraphPad Prism version 8.4.3.

For manuscripts utilizing custom algorithms or software that are central to the research but not yet described in published literature, software must be made available to editors and reviewers. We strongly encourage code deposition in a community repository (e.g. GitHub). See the Nature Research guidelines for submitting code & software for further information.

Data

Policy information about availability of data

All manuscripts must include a data availability statement. This statement should provide the following information, where applicable:

- Accession codes, unique identifiers, or web links for publicly available datasets
- A list of figures that have associated raw data
- A description of any restrictions on data availability

The datasets generated during and/or analysed during the current study are deposited as source files except for patient individualized data sets that underly patient confidentiality restrictions but are available from the corresponding author upon reasonable request. Statistical source data files of the displayed graphs are provided in separate Excel files. Unprocessed immunoblots and agarose gels are provided as source data.
We have provided the doi of the R-code for transcriptomic analyses corresponding to figure (https://doi.org/10.5281/zenodo.7223530). The data set is available upon request as it contains ~omic and, therefore, patient sensitive information.
The same applies to WGS and WES data these we will provide upon request due to data protection issues.
Frome RNAseq data of the patient and controls we exclusively and targetedly extracted the expression information on the genes ASIP, ITCH and AHCY. These data are directly given in supplementary Table S2. Hence, it is not a "classical RNA seq analysis".

Field-specific reporting

Please select the one below that is the best fit for your research. If you are not sure, read the appropriate sections before making your selection.

☒ Life sciences ☐ Behavioural & social sciences ☐ Ecological, evolutionary & environmental sciences

For a reference copy of the document with all sections, see nature.com/documents/nr-reporting-summary-flat.pdf

Life sciences study design

All studies must disclose on these points even when the disclosure is negative.

Sample size	No sample size determination was performed. The manuscript is a case report of a newly identified patient with obesity and novel monogenic mutation in the ASIP gene locus.
Data exclusions	No data was excluded from the study.
Replication	Reproducibility of findings was verified using biological replicates and independent experiments and all exact n values were indicated in figure legends.
Randomization	Randomization was not performed in this study. The manuscript is a case report of a newly identified patient with obesity and novel monogenic mutation in the ASIP gene locus. Wherever possible control patients were mached for age and sex. Details of control patients and control cohorts are given in supplementary table S1 or the respective figure legends.
Blinding	Blinding was not performed in this study. The manuscript is a case report of a newly identified patient with obesity and novel monogenic mutation in the ASIP gene locus. For experimental analyses results were confirmed in several independent experiments. Data collection in cohorts occured not in the scope of this study but before and therefore were blinded.

Reporting for specific materials, systems and methods

We require information from authors about some types of materials, experimental systems and methods used in many studies. Here, indicate whether each material, system or method listed is relevant to your study. If you are not sure if a list item applies to your research, read the appropriate section before selecting a response.

Materials & experimental systems

n/a	Involved in the study
☐	☒ Antibodies
☐	☒ Eukaryotic cell lines
☒	☐ Palaeontology and archaeology
☒	☐ Animals and other organisms
☐	☒ Human research participants
☐	☒ Clinical data
☒	☐ Dual use research of concern

Methods

n/a	Involved in the study
☒	☐ ChIP-seq
☒	☐ Flow cytometry
☒	☐ MRI-based neuroimaging

Antibodies

Antibodies used

anti-ASIP antibody (PA5-77052, Invitrogen, dilution 1:1000); anti-β-actin antibody (ab8227, Abcam, dilution 1:1000)

Validation

Antibodies have been tested by the companies according to their website:
Anti-ASIP antibody: https://www.abcam.com/beta-actin-antibody-ab8227.html
Furthermore, specificity was verified by including positive controls (recombinant ASIP protein), by verifying expected protein size and by performing ASIP knockdown experiments.
Anti-beta Actin: https://www.abcam.com/beta-actin-antibody-ab8227.html

Eukaryotic cell lines

Policy information about cell lines

Cell line source(s)

Human embryo kidney cell line 293 (HEK293) (Sigma-Aldrich, ECACC 85120602),
Chinese hamster ovary cell line CHO-K1 (ATCC CCL-61™),
Human preadipocyte cell line from a patient with Simpson–Golabi–Behmel syndrome (SGBS) provided by Martin Wabitsch (University of Ulm, Germany)
induced pluipotent stem cell lines: HMGU1 and SVF derivied iPSCs (iPSC Core Facility, Helmholtz Zentrum München)

Authentication

None of the cell lines were authenticated.

Mycoplasma contamination

Cell lines were negative for Mycoplasma contamination.

Commonly misidentified lines
(See ICLAC register)

No commonly misidentified cell lines were used in this study.

Human research participants

Policy information about studies involving human research participants

Population characteristics

Detailed characteristics of the study participants are given in the "Case Report" section for the index patient and her father and in the Supplementary Appendix, Table S1 for the control patients. Population characteristics of the control cohorts are provided in the methods section.

Recruitment

The index patient and children of the control group were participants in the Leipzig Adipose Tissue Childhood30 (NCT02208141) and the Leipzig Obesity Childhood (NCT04491344) cohorts.
Control cohorts for obesity were recruited from the outpatient clinic of the university hospital Leipzig and Berlin, lean controls were recruited from the area around Leipzig.

Ethics oversight

Ethics Committee of the University of Leipzig (reg. no. 007-04/027-04, reg. no. 782-1998, reg. no. 029-2006; reg. no. 046-2006, reg. no. 144-10-31052010), Ethics Committee of the Charité – Universitätsmedizin Berlin (EA2/131/11)

Note that full information on the approval of the study protocol must also be provided in the manuscript.

Clinical data

Policy information about clinical studies

All manuscripts should comply with the ICMJE guidelines for publication of clinical research and a completed CONSORT checklist must be included with all submissions.

Clinical trial registration

Leipzig Adipose Tissue Childhood cohort: NCT02208141, Leipzig Obesity Childhood cohort: NCT04491344

Study protocol

Trial protocol can be assessed with the National Clinical Trials accession numbers.

Data collection

Data of the patient were collected between 2005 and now in regular intervals.

Outcomes	The manuscript is a case report of a newly identified patient with obesity and novel monogenic mutation in the ASIP gene locus. Outcomes were diagnostic and obesity-related parameters.
