# Supplementary figures and images for: Aberrant expression of agouti signaling protein (ASIP) as a cause of monogenic severe childhood obesity
Source: Nat Metab. 2022 Dec 19;4(12):1697–712. doi: 10.1038/s42255-022-00703-9 (PMC9771800; doi:10.1038/s42255-022-00703-9)

RAW IMAGE: Fig3E, Cells, ASIP

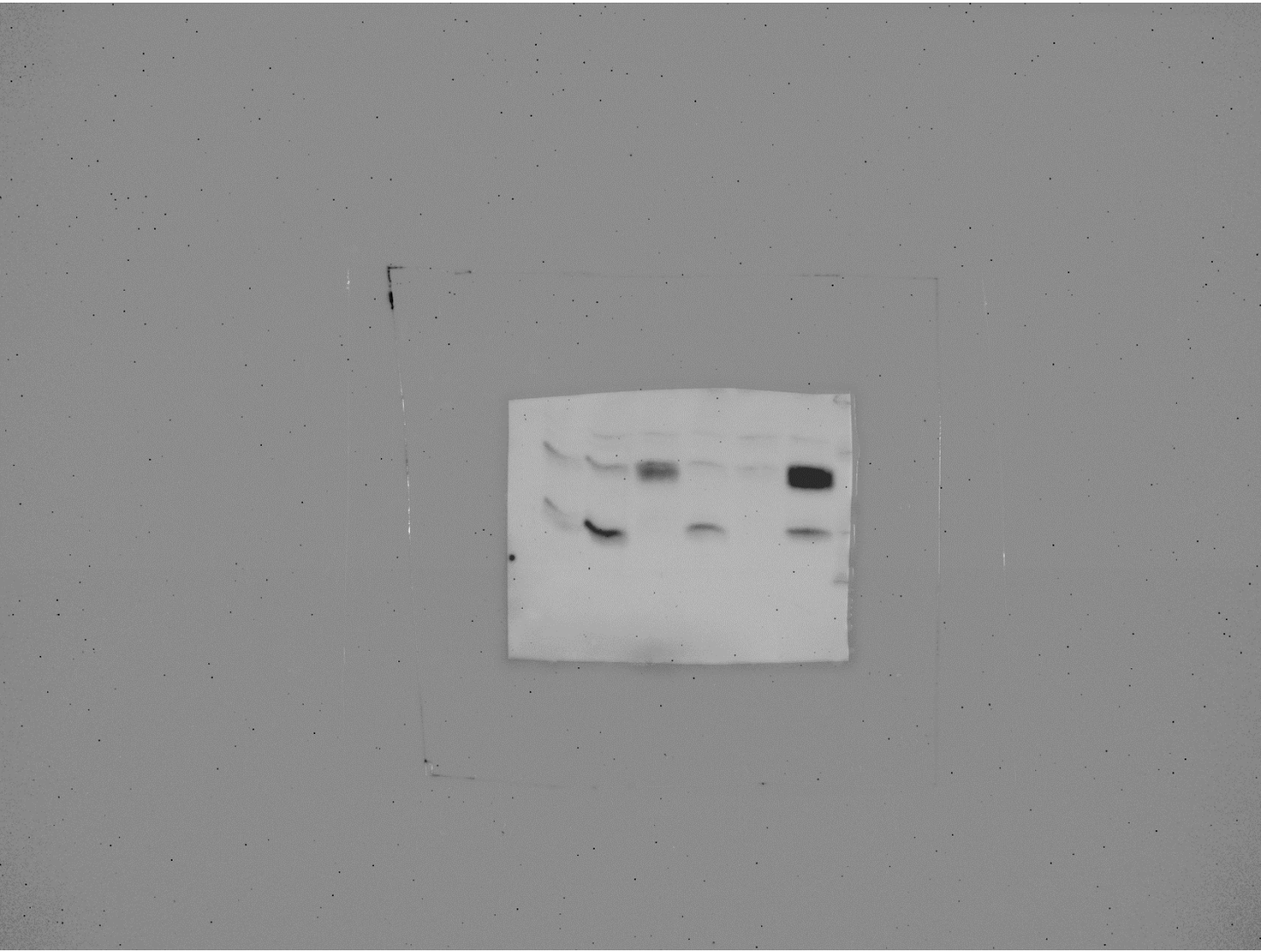

RAW IMAGE: Fig3E, Cells, BACTIN

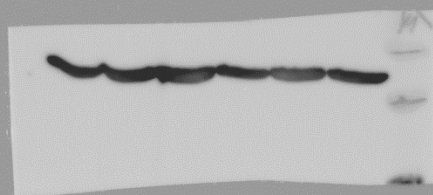

RAW IMAGE: Fig3E, Medium, ASIP

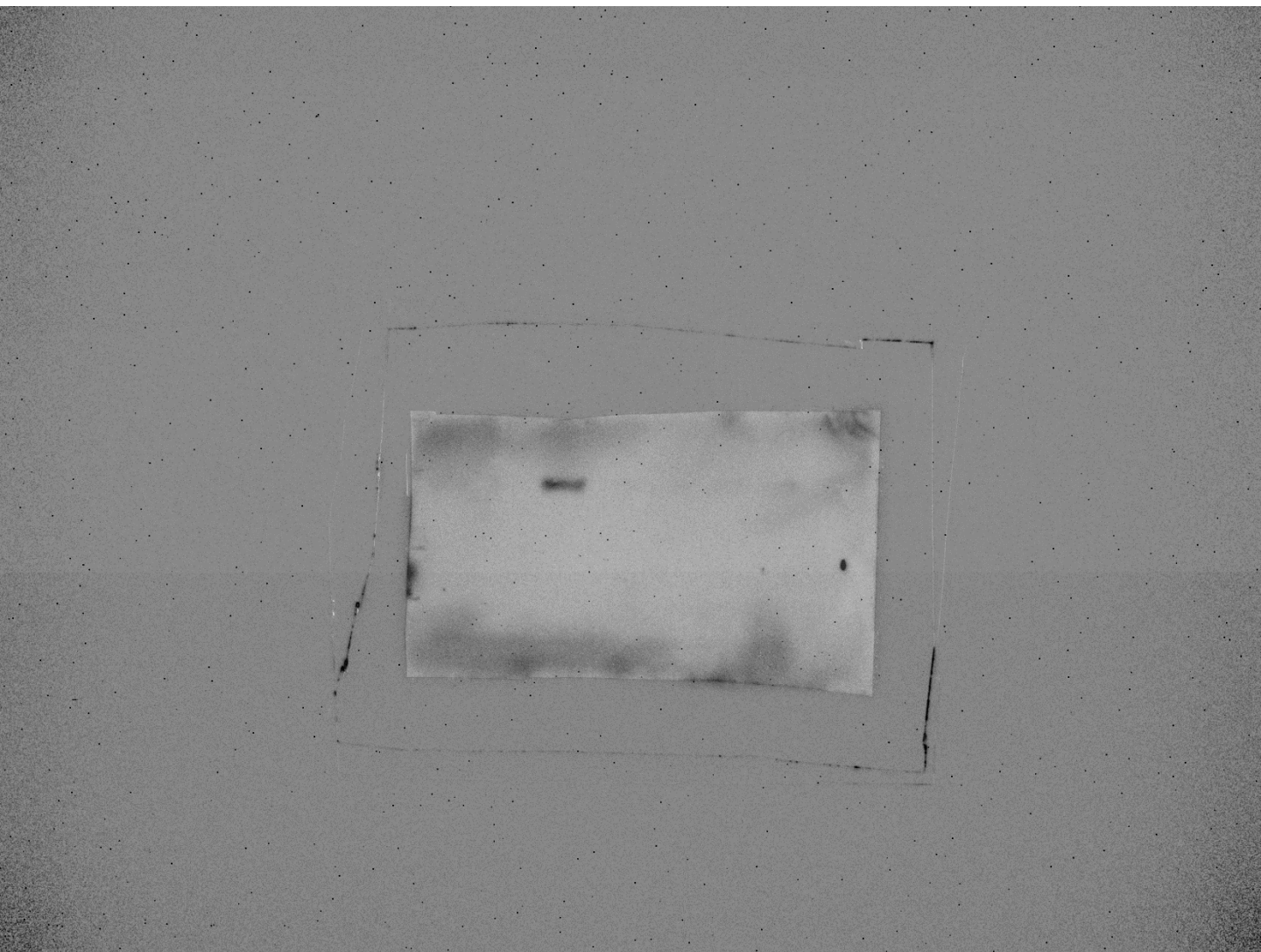

Supplement: Source Data Fig. 3 — Unprocessed and uncropped images of western blots; RAW images Fig. 3. [file 42255_2022_703_MOESM6_ESM.pdf]

RAW IMAGE: Fig5B, Cells, ASIP

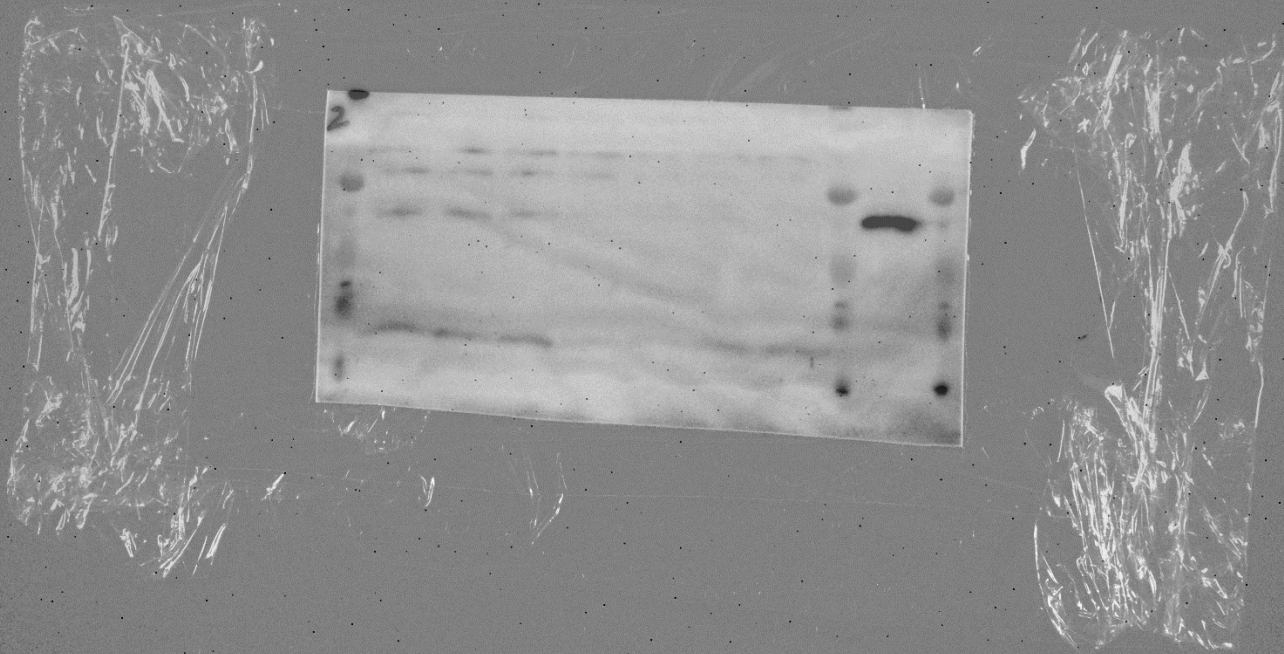

RAW IMAGE: Fig5B, Cells, BACTIN

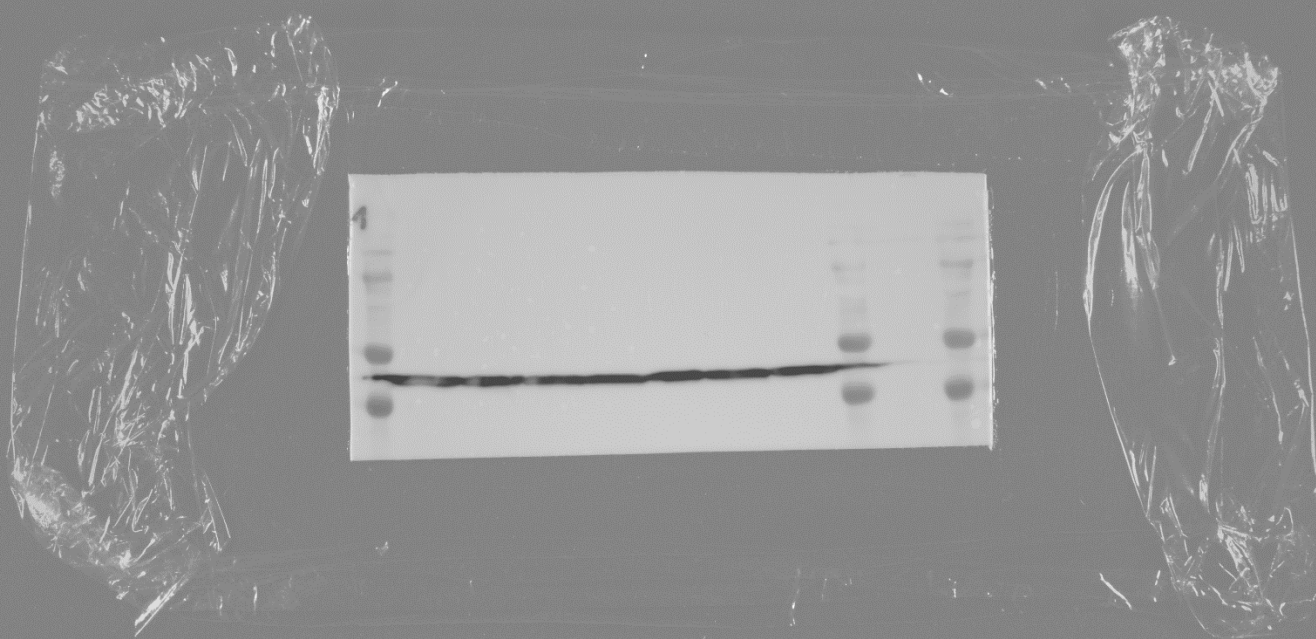

RAW IMAGE: Fig5B, Medium, ASIP

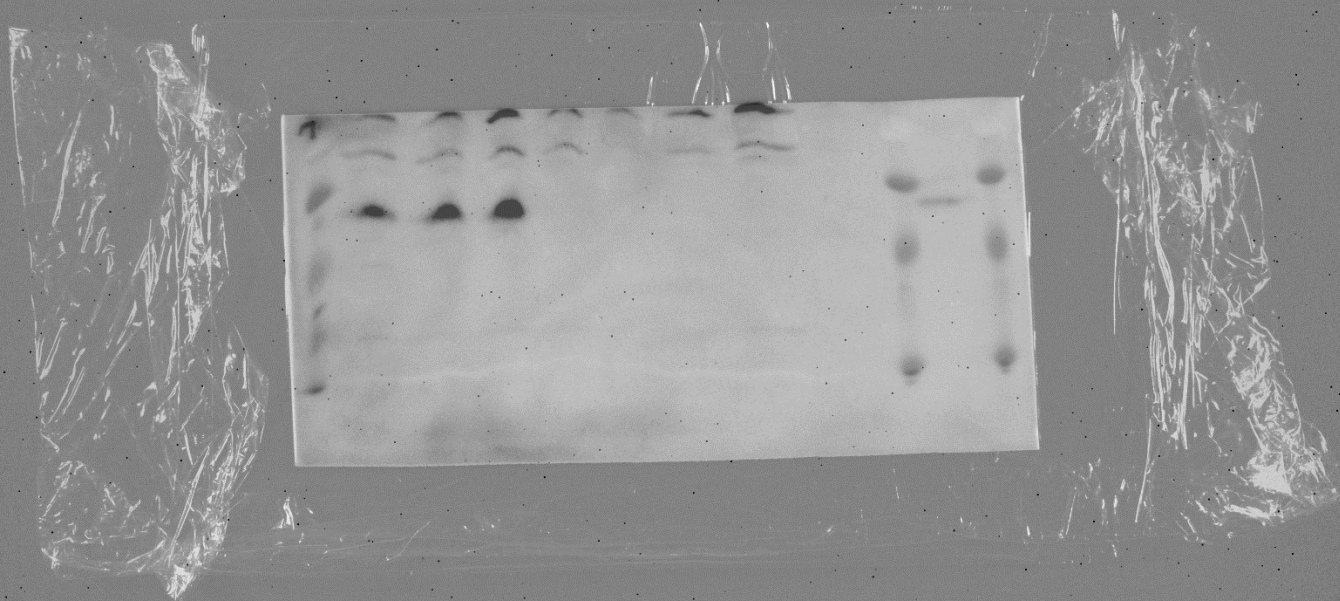

Supplement: Source Data Fig. 5 — Unprocessed and uncropped images of western blots; RAW images Fig. 5. [file 42255_2022_703_MOESM10_ESM.pdf]
