# Supplementary material for: Aberrant expression of agouti signaling protein (ASIP) as a cause of monogenic severe childhood obesity
Source: Nat Metab. 2022 Dec 19;4(12):1697–712. doi: 10.1038/s42255-022-00703-9 (PMC9771800; doi:10.1038/s42255-022-00703-9)

RAW IMAGE: Fig4D

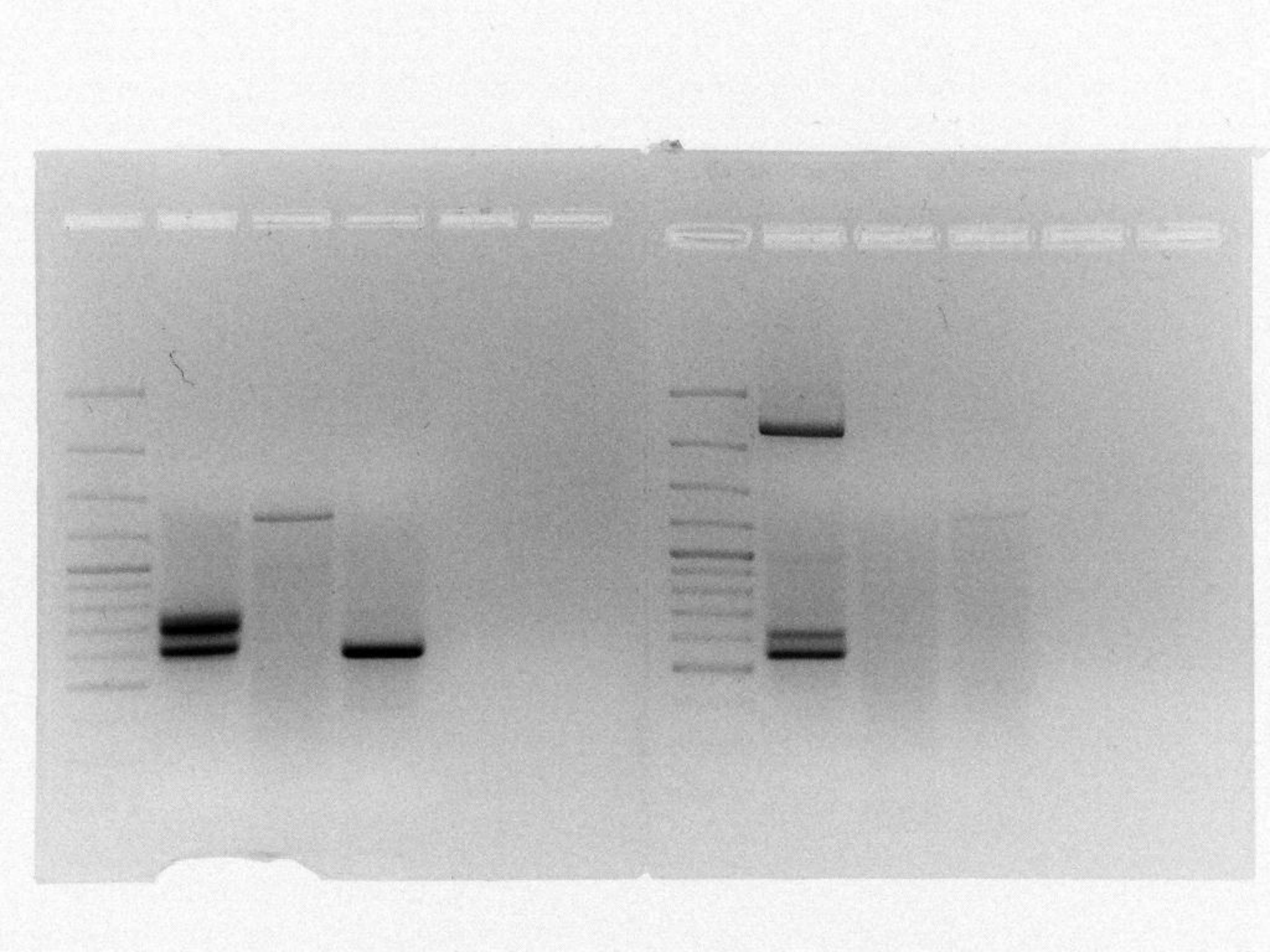

RAW IMAGE: Fig4H, Cells, ASIP

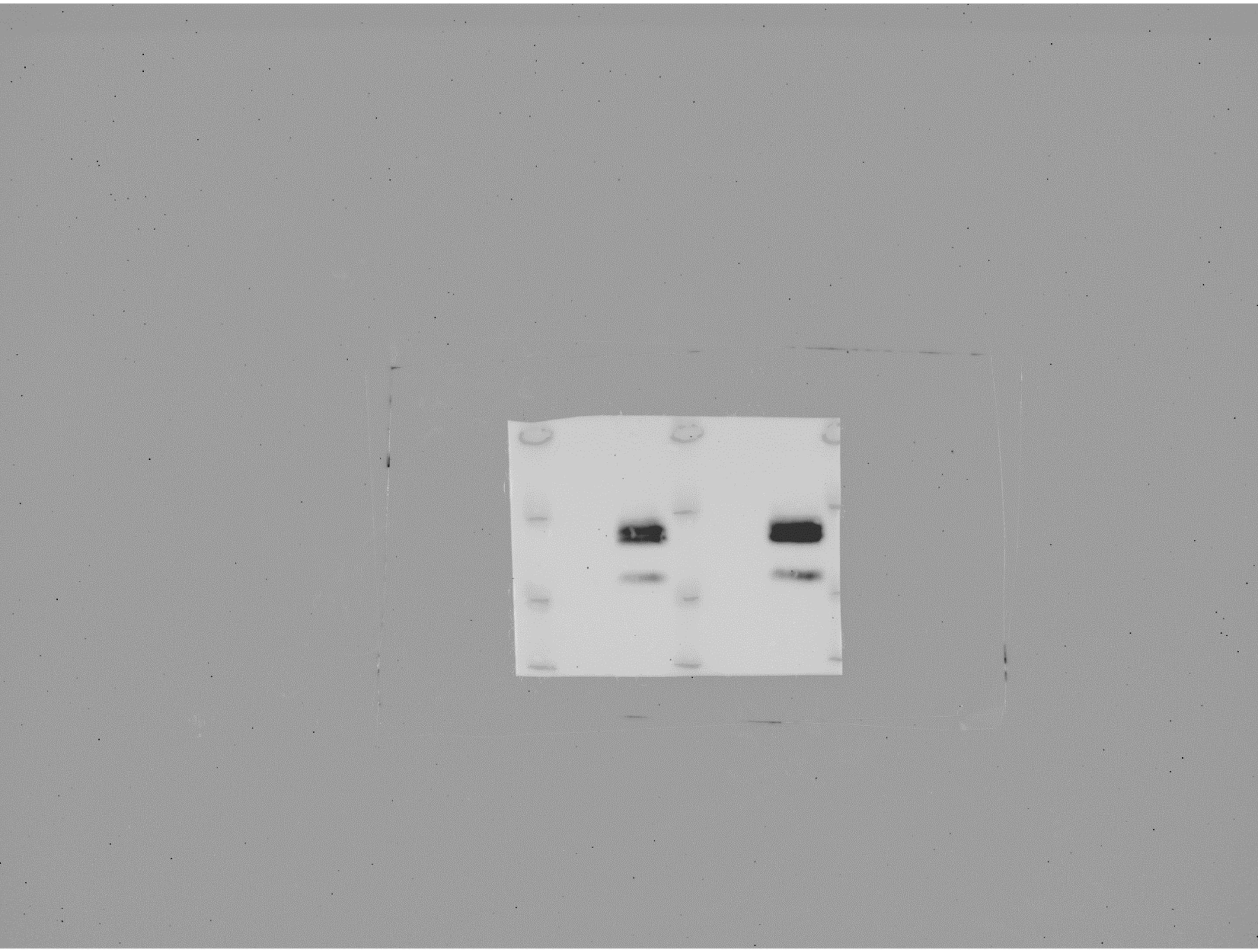

RAW IMAGE: Fig4H, Cells, BACTIN

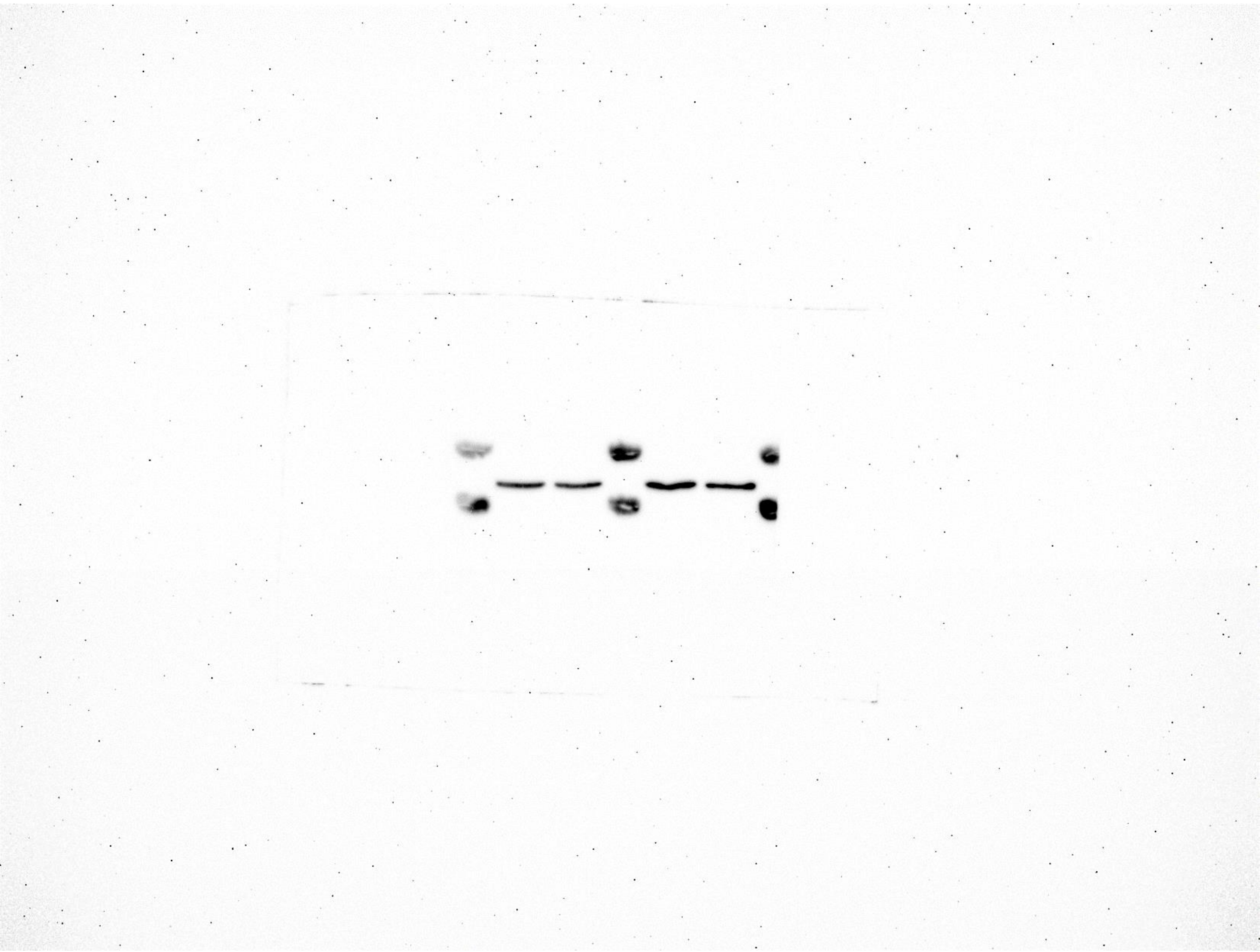

RAW IMAGE: Fig4H, Medium, ASIP

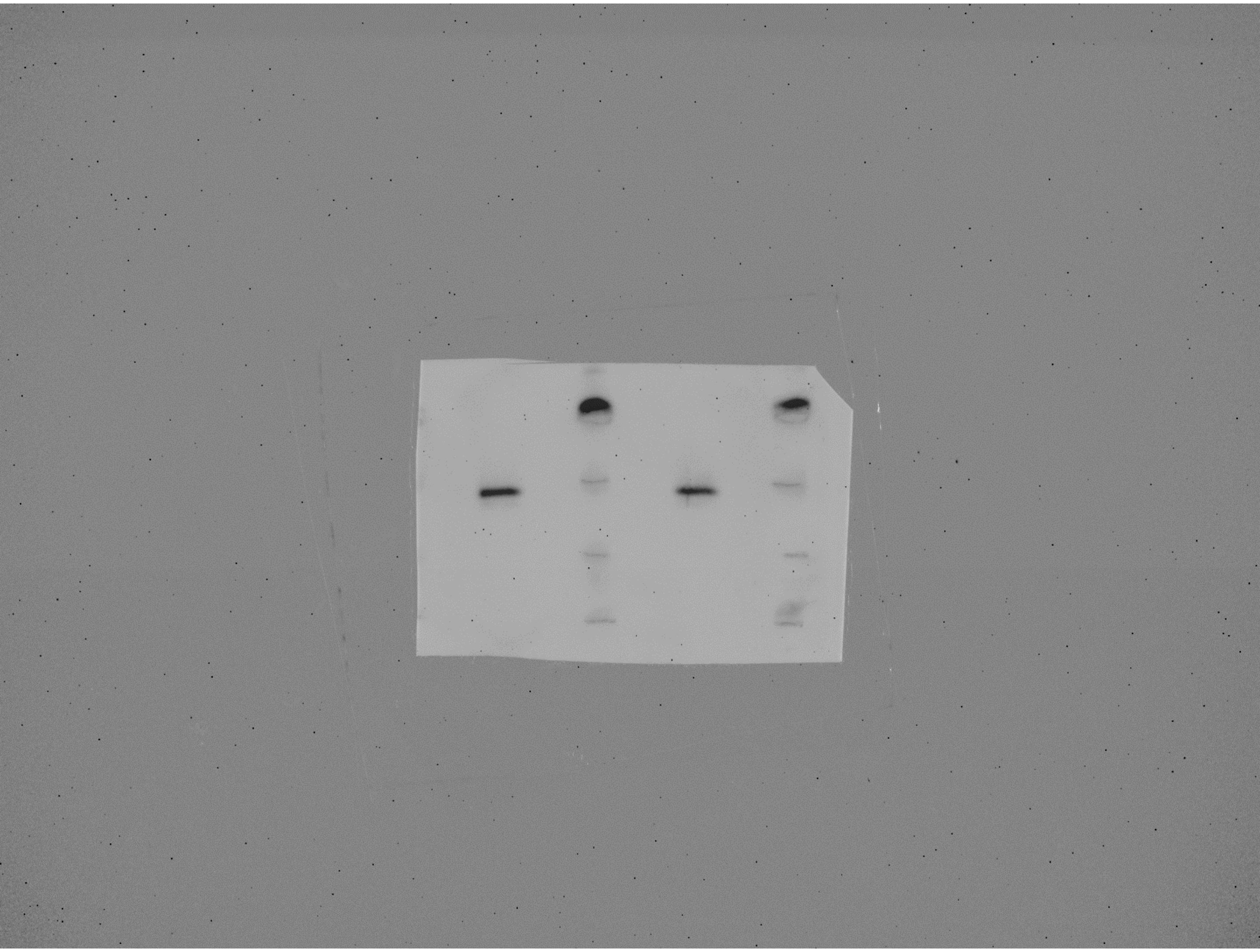

RAW IMAGE: Fig4I, Cells, ASIP

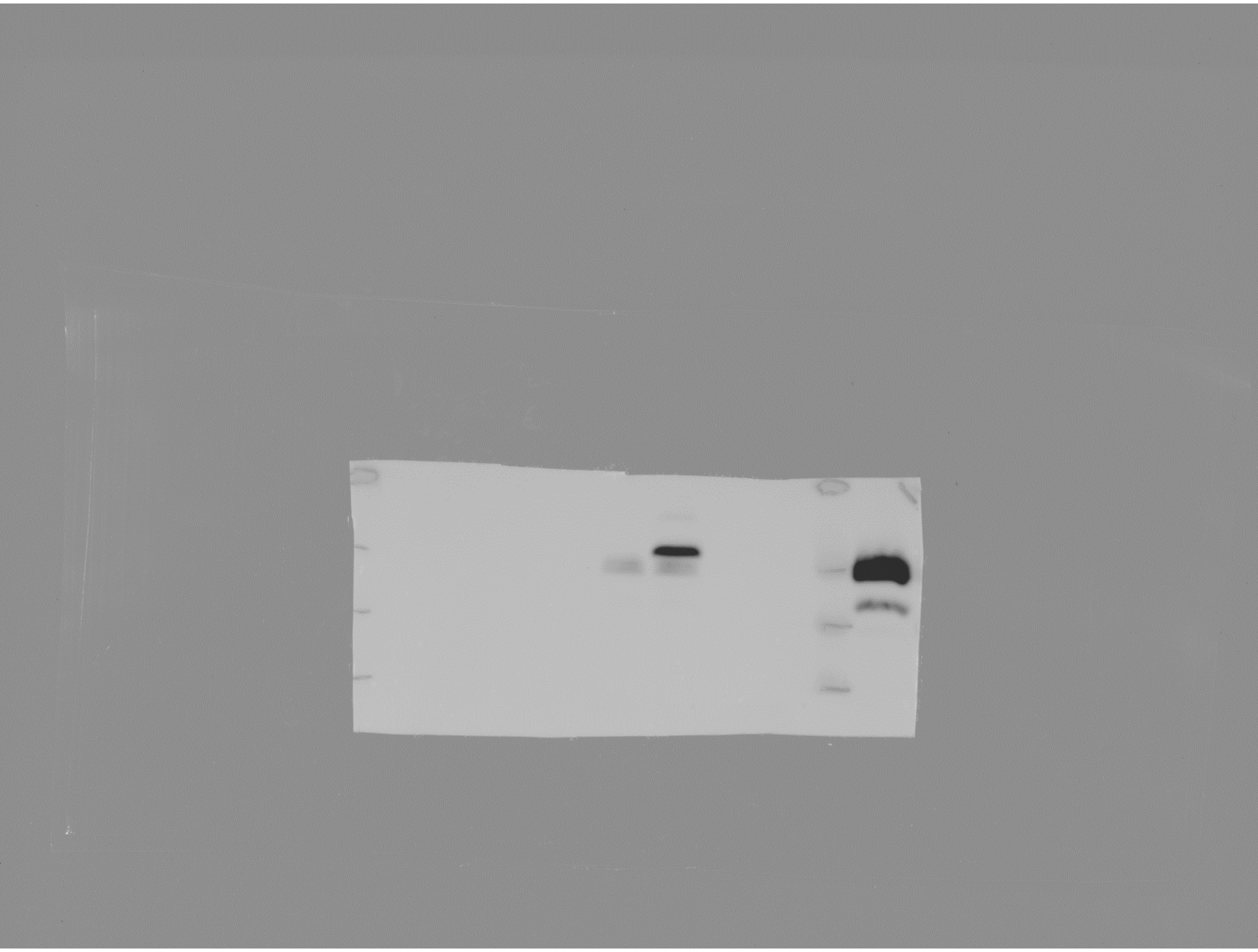

RAW IMAGE: Fig4I, Cells, BACTIN

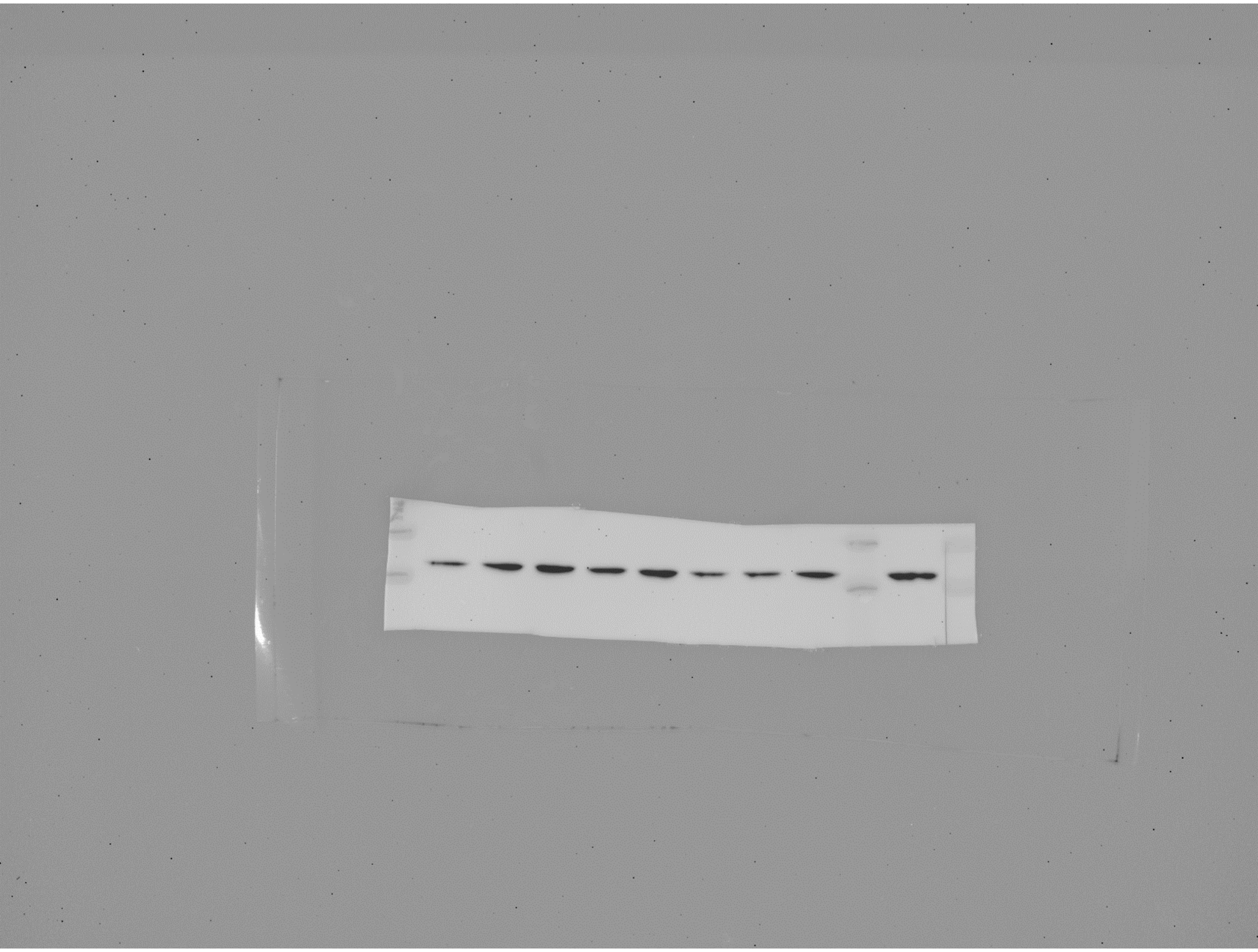

Supplement: Source Data Fig. 4 — Unprocessed and uncropped images of agarose gel and western blots; RAW images Fig. 4. [file 42255_2022_703_MOESM8_ESM.pdf]
